# Highly Efficient Green and Red Narrowband Emissive Organic Light‐Emitting Diodes Employing Multi‐Resonant Thermally Activated Delayed Fluorescence Emitters**

**DOI:** 10.1002/anie.202213697

**Published:** 2022-11-23

**Authors:** Sen Wu, Abhishek Kumar Gupta, Kou Yoshida, Junyi Gong, David Hall, David B. Cordes, Alexandra M. Z. Slawin, Ifor D. W. Samuel, Eli Zysman‐Colman

**Affiliations:** ^1^ Organic Semiconductor Centre EaStCHEM School of Chemistry University of St Andrews Fife KY16 9ST St Andrews UK; ^2^ Organic Semiconductor Centre SUPA School of Physics and Astronomy University of St Andrews KY16 9SS St Andrews UK

**Keywords:** Horizontal Orientation, Hyperfluorescence, Multi-Resonance, Organic Light-Emitting Diodes, Thermally Activated Delayed Fluorescence

## Abstract

Herein, we demonstrate how judicious selection of the donor decorating a central multi‐resonant thermally activated delayed fluorescence (MR‐TADF) core based on DiKTa can lead to very high‐performance OLEDs. By decorating the DiKTa core with triphenylamine (TPA) and diphenylamine (DPA), **3TPA‐DiKTa** and **3DPA‐DiKTa** exhibit bright, narrowband green and red emission in doped films, respectively. The OLEDs based on these emitters showed record‐high performance for this family of emitters with maximum external quantum efficiencies (EQE_max_) of 30.8 % for **3TPA‐DiKTa** at λ_EL_ of 551 nm and 16.7 % for **3DPA‐DiKTa** at λ_EL_=613 nm. The efficiency roll‐off in the OLEDs was improved significantly by using 4CzIPN as an assistant dopant in hyperfluorescence (HF) devices. The outstanding device performance has been attributed to preferential horizontal orientation of the transition dipole moments of **3TPA‐DiKTa** and **3DPA‐DiKTa**.

## Graphical abstract

Through careful selection of donor substituents about the DiKTa core, record‐high efficiency green‐emitting OLEDs were fabricated, with EQE_max_ of 30.8 %, and the reddest MR‐TADF OLEDs have been reported based on ketone‐containing multi‐resonant thermally activated delayed fluorescent emitters.

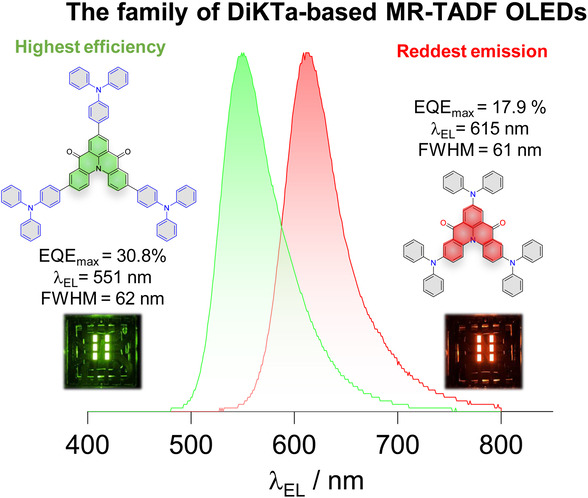

## Introduction

Organic light‐emitting diodes (OLEDs) have emerged as an exciting display technology that has steadily gained market share in a number of consumer electronic markets, from mobile phones and smart watches to televisions and monitors. The quality of an OLED depends on three key factors: its stability, its efficiency and its color purity. All of these parameters are linked in part to the intrinsic properties of the emitter. Two strategies to achieve high color purity in the device are to (1) employ a narrowband emissive material and/or (2) employ color filters to select out the desired emission. The use of color filters leads inevitably to light being lost by absorption, and a lower efficiency device,^[1]^ thus there is a strong desire to develop narrowband emitters. A high‐efficiency device also requires the use of emitter materials that can efficiently convert both singlet and triplet excitons into light. Two classes of emitters are capable of realizing 100 % internal quantum efficiency (IQE) in the device, these are phosphorescent compounds and compounds that emit via thermally activated delayed fluorescence (TADF). Organic TADF emitters harvest both singlet and triplet excitons via an endothermic reverse intersystem crossing (RISC) process that converts non‐emissive triplet excitons into singlets prior to light generation.^[2]^ RISC is enabled when the energy gap between the lowest‐lying singlet and triplet excited states (Δ*E*_ST_) is sufficiently small.^[3]^ A molecular design that shows a small exchange integral between molecular orbitals related to the excited state is responsible for the small Δ*E*_ST_ in the emitter. This is typically accomplished using a twisted structure containing donor (D) and acceptor (A) fragments.^[3d]^ However, the charge‐transfer (CT) excited state[^2b^, ^4^] and the large reorganization energy in the excited state lead to broad emission, characterized by large full width half‐maximum (FWHM) >80 nm.[^3c^, ^5^] The corresponding TADF OLEDs do not show the desired high color purity.

A solution is to use multi‐resonant TADF (MR‐TADF) emitters.^[6]^ MR‐TADF emitters are typically based on p‐ and n‐doped nanographenes and so possess a rigid molecular structure.^[7]^ The ingenious molecular design places each of the frontier molecular orbital (FMOs) on adjacent atoms thus leading to short‐range charge transfer (SRCT) between adjacent atoms, inducing the small Δ*E*_ST_ to turn on TADF. The SRCT excited state that results from the transition from the HOMO to the LUMO coupled with the small reorganization energy result in narrowband emitters. Since the first examples of this class of emitter, documented in 2016 by Hatakeyama and co‐workers, more than 200 distinct examples have been reported, most of which show blue and green emission.^[3a]^ We previously demonstrated that decorating a MR‐TADF core with peripheral donor groups can result in emission tuning.^[8]^ However, when too strong donors are employed then the lowest energy excited states are no longer SRCT and the narrowband emission is lost. Rather, the emissive excited state becomes long‐range CT (LRCT) that is typically observed in donor‐acceptor TADF compounds. The aforementioned reports evidence of emission tuning from 462 to 608 nm using a peripheral decoration strategy on the MR‐TADF core; nevertheless, examples of red‐emitting MR‐TADF emitters remain rare.

**Figure 1:**
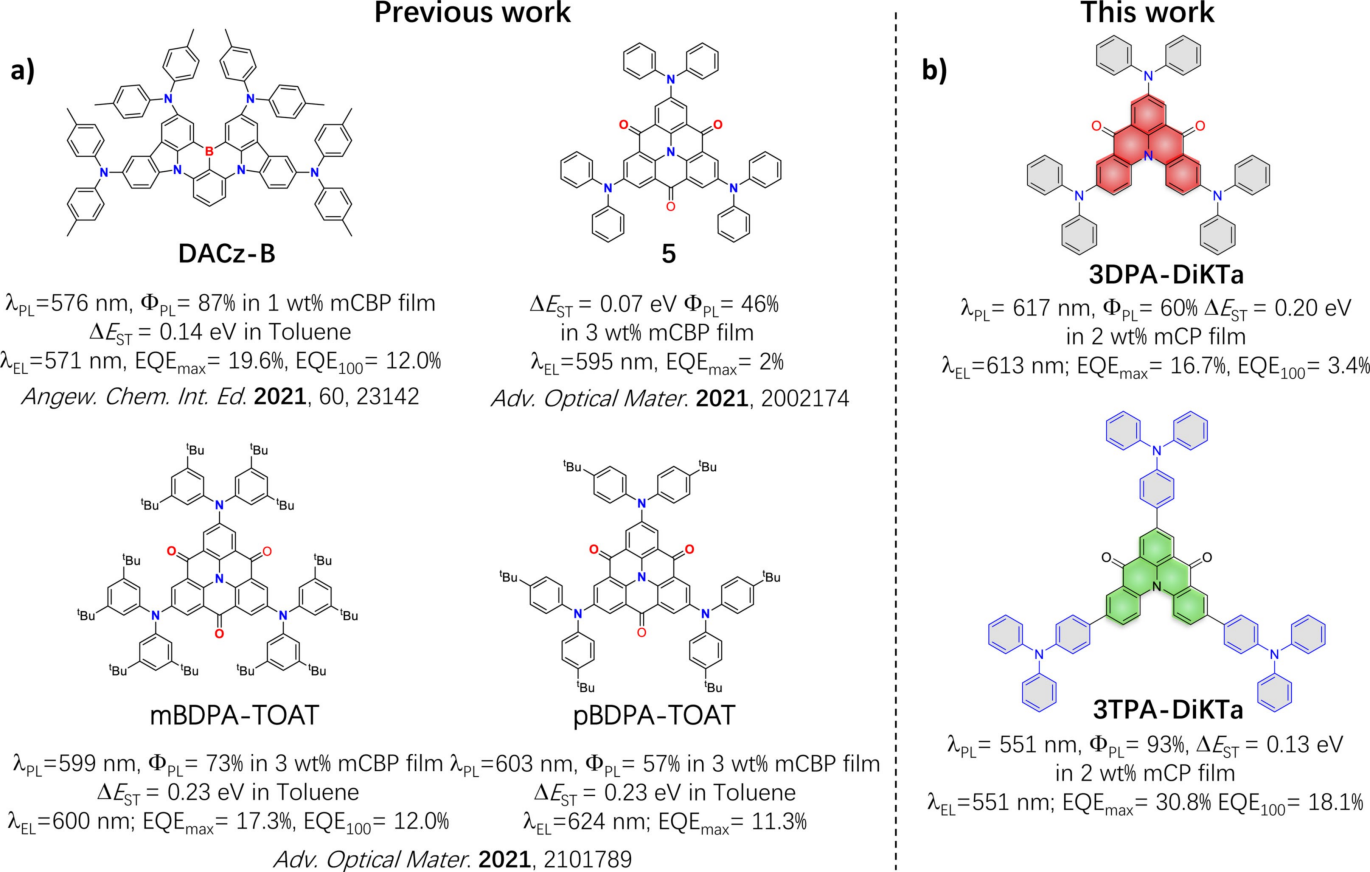
a) Chemical structures, photophysical and OLED data of reported MR‐TADF materials containing DPA units. b) Chemical structures, photophysical and OLED data of **3DPA‐DiKTa** and **3TPA‐DiKTa** of this work.

Fluorobenzene,^[9]^ *tert*‐butylcarbazole,^[10]^ *tert*‐butylbenzene,^[10a]^ and cyanobenzene^[11]^ are the most frequently used substituents to decorate MR‐TADF emitters. These examples are either weak electron‐withdrawing and/or electron‐donating groups and so their incorporation does not disrupt the MR‐TADF character. Diphenylamine (DPA) possesses an intermediate electron‐donating ability between carbazole, which allows retention of MR‐TADF when it is added to a core, and dimethylacridan DMAC, which results in a D‐A emitter when it is decorated about a MR‐TADF core. The incorporation of peripheral DPA groups has been shown to red‐shift the emission of MR‐TADF compounds by Yasuda and co‐workers. For instance, **DACz‐B** (Figure 1) shows bright yellow narrowband emission^[12]^ (photoluminescence emission peak wavelength, λ_PL_=576 nm; full width half‐maximum (FWHM)=44 nm; photoluminescence quantum efficiencies (Φ_PL_) of 87 %), which is red‐shifted compared to **Cz‐B** (λ_PL_=484 nm; FWHM=30 nm; Φ_PL_ of 97 %, Figure S3) in 1 wt %‐doped 3,3′‐Di(9*H*‐carbazol‐9‐yl)‐1,1′‐biphenyl (mCBP) film. The OLED with **DACz‐B** produces yellow electroluminescence (peak electroluminescence wavelength, λ_EL_=571 nm) with a maximum external EL quantum efficiency (EQE_max_) of 19.6 %. Using a similar room temperature phosphorescence skeleton, **TOAT**, Adachi and co‐workers introduced different numbers of DPA groups *para* to the central nitrogen atom of the **TOAT** core (**3**, **4** and **5**, see Figure S3).^[13]^ This led to the realization of red emission with a FWHM 45 nm in toluene for emitter **5**. Using the same **TOAT** core, Zhang and co‐workers reported DPA analogues **mBDPA‐TOAT** and **pBDPA‐TOAT** ^[14]^ that contained *tert*‐butyl groups on the periphery of the DPA groups (Figure 1), which showed orange emission of 571 (FWHM=34 nm) and 563 nm (FWHM=48 nm) in toluene, respectively. However, the OLEDs using a TOAT derivative typically show low device performance with EQE_max_<18 %. We recently reported a study wherein the MR‐TADF OLEDs using **3Cz‐DiKTa** as the emitter showed a λ_EL_ of 547 nm (FWHM=54 nm) and an EQE_max_ of 24.4 % (Figure S3 and Table S9).^[8]^ This initial study revealed the fine balance that must be achieved when decorating MR‐TADF emitters with peripheral donors.

Here we report two emitters containing either three diphenylamine (DPA) or triphenylamine (TPA) donor groups decorating a central MR‐TADF core, **DiKTa**: **3DPA‐DiKTa** and **3TPA‐DiKTa** (Figure 1). Both emitters show a desired red‐shifted emission compared with **3Cz‐DiKTa** ^[8]^ and behave as MR‐TADF compounds, characterized by small Δ*E*_ST_, narrow FWHM, and high Φ_PL_ in 1,3‐bis(*N*‐carbazolyl)benzene (mCP) doped films. **3TPA‐DiKTa** shows an emission maximum at λ_PL_=551 nm (FWHM=58 nm) with Φ_PL_=93 % while the emission of **3DPA‐DiKTa** is red‐shifted (λ_PL_=617 nm, FWHM=56 nm, Φ_PL_=60 %) in the 2 wt % mCP doped films. Benefitting from both the high Φ_PL_ and the enhanced light‐outcoupling associated with a preferential horizontal orientation of its transition dipole moment, the OLED with **TPA‐DiKTa** exhibits an EQE_max_=30.8 % at a λ_EL_ of 551 nm (FWHM of 62 nm) and the device with **3DPA‐DiKTa** shows an EQE_max_ of 16.7 % with a λ_EL_ of 615 nm (FWHM of 60 nm). The efficiency roll‐off was significantly improved from 41 % to 8.6 % for the OLED with **3TPA‐DiKTa** and from 80 % to 51 % for the OLED with **3DPA‐DiKTa** at 100 cd m^−2^ by using 1,2,3,5‐tetrakis(carbazol‐9‐yl)‐4,6‐dicyanobenzene, 2,4,5,6‐tetrakis(9*H*‐carbazol‐9‐yl) isophthalonitrile (4CzIPN) as an assistant dopant in hyperfluorescence (HF) devices. This work demonstrates how molecular engineering can lead to the most efficient ketone‐containing MR‐TADF emitters discovered so far.

## Results and Discussion

The synthesis routes to **3DPA‐DiKTa** and **3TPA‐DiKTa** are shown in Schemes S1–S2. The previously reported intermediate 3Br‐DiKTa^[7]^ was elaborated with either diphenylamine or triphenylamine donors via a three‐fold palladium‐catalyzed Buchwald–Hartwig or Suzuki–Miyaura cross‐coupling reaction, respectively. The identity and purity of the two emitters were determined using a combination of ^1^H and ^13^C NMR spectroscopy, high‐resolution mass spectrometry, element analysis, high‐pressure liquid chromatography (HPLC), single‐crystal‐XRD and melting point determination (Figures S10–S18).

**Figure 2:**
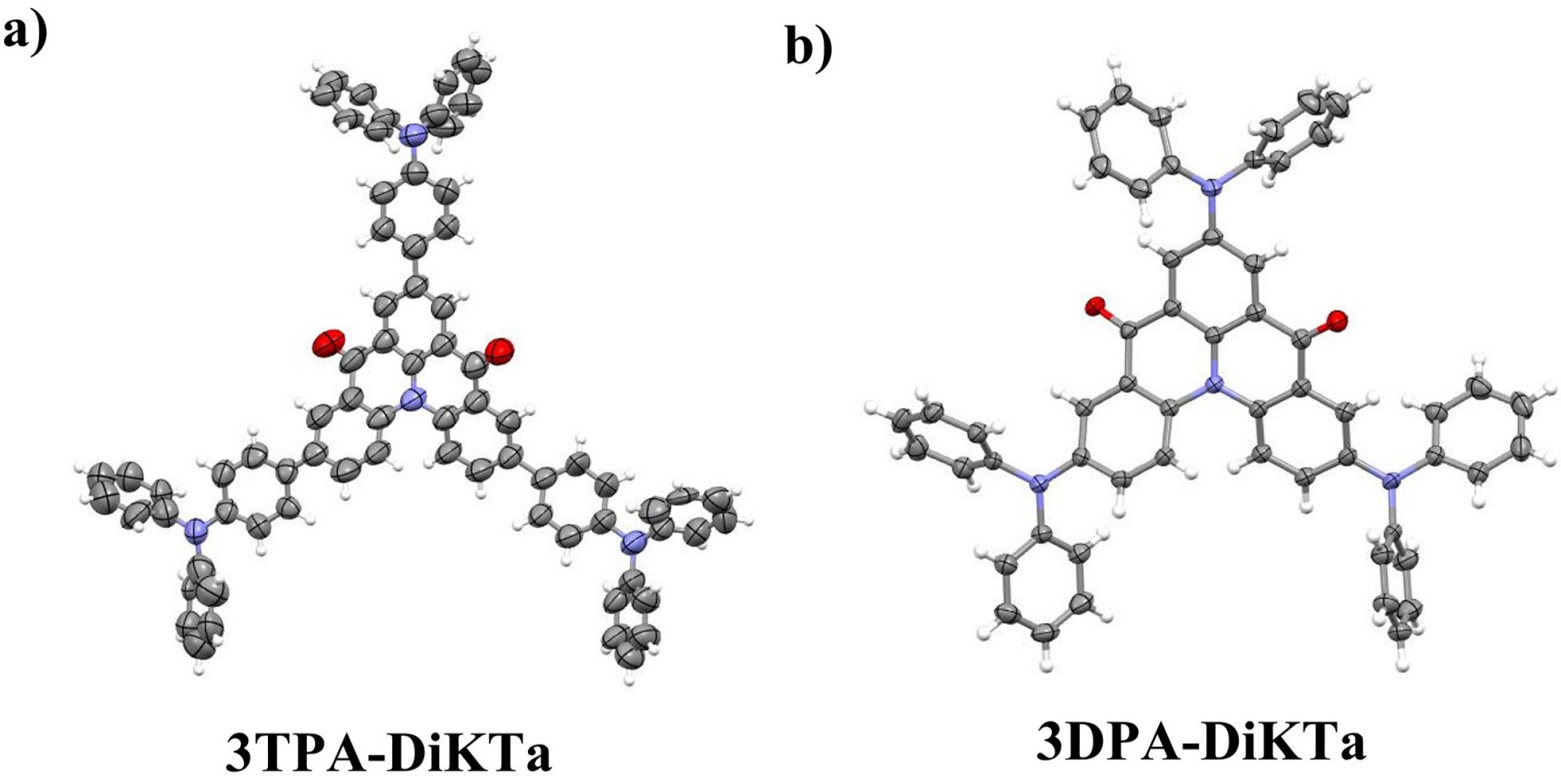
Thermal ellipsoid plot of the crystal structure of a) **3TPA‐DiKTa** (CCDC number: 2183420) and b) **3DPA‐DiKTa** (CCDC number: 2183421). Ellipsoids are drawn at the 50 % probability level and solvent molecules; minor components of disorder are omitted for clarity.

Single crystals of **3TPA‐DiKTa** and **3DPA‐DiKTa** (Figure 2 and Table S1) suitable for X‐ray diffraction were both obtained through slow liquid diffusion of hexane in a DCM solution at room temperature. The DiKTa core in both emitters showed a low degree of planarity because of the presence of the puckered acridone moieties, fused with a common nitrogen. The degrees of puckering between the two emitters are somewhat similar, with angles between the common phenyl ring plane and the peripheral ring planes in the fused acridone of 13.96° and 17.09° in **3TPA‐DiKTa** and 15.85° and 27.80° in **3DPA‐DiKTa**. In **3TPA‐DiKTa** the bridging phenylene rings are close to co‐planar with the DiKTa core. The degree of planarity of the geometry of both molecules was then determined using DFT at the PBE0/6‐31G(d,p) level based on the crystal structures as the starting geometry. Overall, both molecules show planarity ratios of 67 % for **3TPA‐DiKTa** 68 % for **3DPA‐DiKTa** (Figure S5), indicating that the compounds in the crystal structure geometry are reasonably planar.

**Figure 3:**
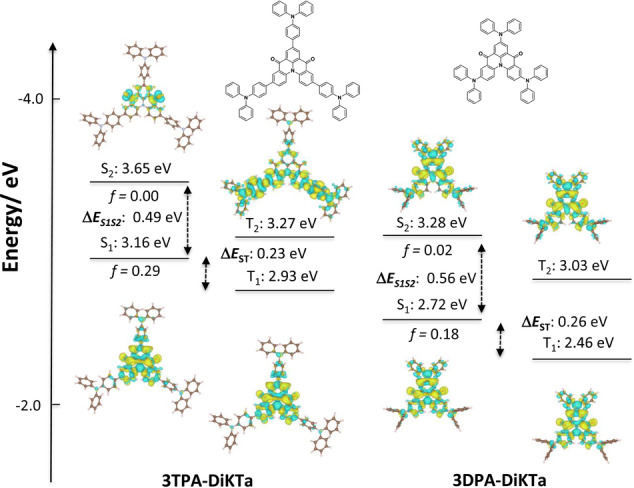
Difference density plots of S_1_/S_2_ and T_1_/T_2_ excited states (calculated in the gas phase at the SCS‐ADC(2)/cc‐pVDZ level) for **3TPA‐DiKTa** and **3DPA‐DiKTa**. *f* is the oscillator strength.

The nature of the lowest‐energy excited states can play a significant role in the color purity of the emitter. In particular, the narrowband of MR‐TADF emitters is conserved when the S_1_ state possesses SRCT, whereas the emission can broaden in some cases when the MR‐TADF core is decorated with donor groups due to the stabilization of the LRCT state that dominates in donor‐acceptor systems.^[8]^ To gain insight into the relative energies of these states and to rationalize the optoelectronic properties of the emitters, the FMOs were first modelled at the optimized ground state geometry in the gas‐phase using the density functional theory (DFT) at the PBE0/6‐31G(d,p) level of theory. In contrast to the planarity values calculated from the X‐ray structures, those calculated from the DFT‐optimized structures show planarity differences between the two compounds (Figure S6). **3TPA‐DiKTa** adopts a more planar conformation, with a planarity ratio of 74 %, compared to **3DPA‐DiKTa** where the planarity ratio is 42 %. According to Figure S7, the LUMOs for both molecules are only distributed across the DiKTa core. Due to the introduction of the peripheral donors, the LUMOs of **3TPA‐DiKTa** and **3DPA‐DiKTa** are destabilized at −2.13 and −2.09 eV compared to that of **DiKTa** (−2.23 eV). The HOMO of **DPA‐DiKTa** is distributed across the entire molecule while the HOMO is mainly located on the peripheral TPA units in **3TPA‐DiKTa**, which implies that this latter compound should possess a low‐lying LRCT state (Figure S7). The HOMO values (*E*_HOMO_) for **3TPA‐DiKTa** and **3DPA‐DiKTa** are −5.11 eV and −5.00 eV, respectively, which are significantly destabilized compared to that of **DiKTa** (*E*_HOMO_=−6.02 eV). The shallower HOMO and LUMO values for **3DPA‐DiKTa** compared with **3TPA‐DiKTa** are attributed to the stronger electron‐donating strength of DPA than TPA. The corresponding HOMO–LUMO gaps for **3DPA‐DiKTa** and **3TPA‐DiKTa** are of 2.91 and 2.98 eV, respectively. We have previously shown that DFT methods are not suitable for the accurate modelling of the excited states of MR‐TADF compounds.^[15]^ We thus proceeded to model the excited states using the second order algebraic diagrammatic construction (ADC(2)) with the cc‐pVDZ basis set using the spin component scaling (SCS‐) approximation. The different density plots of the low‐lying singlet and triplet excited states are shown in Figure 3. The S_1_ difference densities of both emitters show similar patterns to that of **DiKTa** (Figures S4 and S7). Based on the charge‐transfer distance, D_CT_ <0.6 Å, we assign these excited states to be SRCT (Table S3). For both emitters, there are small contributions to the difference density from the peripheral donors. Specifically, the difference density plot of **3DPA‐DiKTa** indicates that the electron density is delocalized to the nitrogen atoms of DPA group, which will increase the electron‐donating strength, contributing to a more stabilized S_1_ state, while the decreased difference density of **3TPA‐DiKTa** is mostly located on the phenyl rings of the TPA groups, thereby having less of an influence on the energy of the S_1_ state relative to the DPA groups in **3DPA‐DiKTa**. The presence of the phenylene bridge between the DPA groups and the DiKTa core in **3TPA‐DiKTa** reduces the HOMO and LUMO overlap but also increases the oscillator strength (*f*) in **3TPA‐DiKTa** in comparison to **3DPA‐DiKTa** (0.29 vs. 0.18, respectively). The S_2_ state of **3TPA‐DiKTa** has a typical n‐π^*^ character, while for **3DPA‐DiKTa** S_2_ has similar difference density to S_1_. According to our previous study, the SRCT should dominate the S_1_ emission process under a low polarity environment.[^8^, ^16^] The predicted S_1_/T_1_ energies for **3TPA‐DiKTa** and **3DPA‐DiKTa** are 3.16/2.93 and 2.72/2.46 eV, respectively, with corresponding Δ*E*_ST_ values of 0.23 eV and 0.26 eV, which is similar to **DiKTa** (0.27 eV).

**Figure 4:**
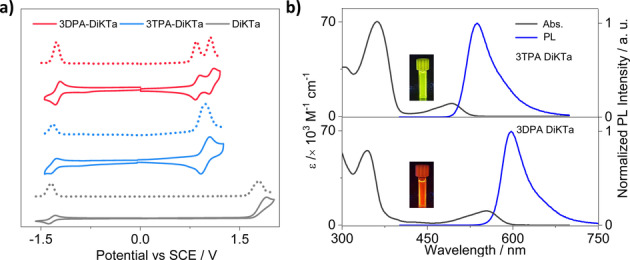
a) Cyclic voltammogram (CV) and differential pulse voltammetry (DPV) in degassed DCM with 0.1 M [*n*Bu_4_N]PF_6_ as the supporting electrolyte and Fc/Fc^+^ as the internal reference (0.46 V vs. SCE).^[21]^ b) Absorption and steady‐state PL spectra (SS) obtained in toluene at room temperature (λ_exc_=340 nm). The inset are photographs of the photoluminescence from the toluene solution.

Cyclic voltammetry (CV) and differential pulse voltammetry (DPV) measurements were carried out in dichloromethane (DCM) to experimentally ascertain the HOMO and LUMO levels (Figure 4a). The electrochemical data is compiled in Table S4. The reduction waves of **3TPA‐DiKTa** and **3DPA‐DiKTa** are reversible and almost identical in potential to that of **DiKTa**. Based on the DFT calculations, the reduction is localized on the DiKTa core. The calculated LUMO levels are thus similar at −2.98 eV for **3TPA‐DiKTa** and −3.01 eV for **3DPA‐DiKTa** to at −3.00 eV for **DiKTa**. Both emitters also show reversible oxidation waves, which correspond to the oxidation of the peripheral amine donor units; **3DPA‐DiKTa** shows two oxidation waves, reflecting unbalanced holes density distribution from the two electrochemically distinct DPA groups. The HOMO values of both emitters are destabilized at −5.13 eV for **3DPA‐DiKTa** and −5.27 eV for **3TPA‐DiKTa** compared to that of **DiKTa** at −6.12 eV. The trend in HOMO levels aligns with the DFT calculations (Table S2). The corresponding electrochemical gap for **3DPA‐DikTA** is 2.12 eV and for **3TPA‐DiKTa** is 2.29 eV, both significantly smaller than that of **DiKTa** at 3.12 eV.

Spectroscopic measurements (absorption and photoluminescence) in dilute toluene (10^−5^ M) at room temperature were undertaken to understand the photophysical properties of the monomolecular species. The spectra are shown in Figure 4b. For both emitters the absorption band below 400 nm is attributed to a locally excited (LE) π–π* transition of the whole skeleton.^[17]^ The longer wavelength SRCT absorption bands at 494 nm for **3TPA‐DiKTa** and 554 nm for **3DPA‐DiKTa** are red‐shifted compared to that of **DiKTa** at 433 nm. The PL maxima of **3DPA‐DiKTa** occurs at 597 nm and **3TPA‐DiKTa** occurs at 537 nm, both significantly red‐shifted compared to that of **DiKTa** (λ_PL_=453 nm); indeed, **3DPA‐DiKTa** shows the reddest emission among the DiKTa‐based MR emitters.[^13^, ^14^, ^18^] The Stokes shift of **3TPA‐DiKTa** is 42 nm and of **3DPA‐DiKTa** is 41 nm. The emission of both emitters is narrow, with FWHM of 54 nm or energy width at half maxima (EWHM) of 252 meV for **3TPA‐DiKTa** and FWHM of 47 nm or EWHM of 168 meV for **3DPA‐DiKTa**. These values reveal only a modest degree of reorganization in the excited state compared to the ground state. The slightly broader emission of **3TPA‐DiKTa** than **3DPA‐DiKTa** is due to a greater admixture of CT character to the SRCT emissive excited state. The EWHM value of **3DPA‐DiKTa** is smaller than that of **3TPA‐DiKTa** yet similar to that of **DiKTa** (172 meV or 27 nm), indicating the more pronounced LRCT character of excited state of **3TPA‐DiKTa**, which aligns with the coupled cluster calculations analysis (Figure S22b).^[19]^ Figure S19 shows the effect of oxygen on the steady‐state PL in toluene. The Φ_PL_ values in degassed toluene are 44 % for **3TPA‐DiKTa** and 59 % for **3DPA‐DiKTa**, which decreased in the presence of oxygen to 33 % for **3TPA‐DiKTa** and 38 % for **3DPA‐DiKTa**, indicating that triplets play a significant role in the light emission process.^[20]^ The time‐resolved PL in degassed toluene has mono‐exponential decay kinetics for both compounds, with PL lifetimes, τ_PL_, of 11 ns for **3TPA‐DiKTa** and 19 ns for **3DPA‐DiKTa**, (Figure S20); no delayed emission was detected despite the oxygen sensitivity noted for the Φ_PL._ Thus, the contribution from the delayed fluorescence in **3TPA‐DiKTa** and **3DPA‐DiKTa** in toluene is smaller than we can reliably identify.

We next determined the singlet/triplet (S_1_/T_1_) energies of both emitters in 2‐MeTHF glass at 77 K (Figure S21). The S_1_/T_1_ energies are 2.48/2.27 eV for **3TPA‐DiKTa** and 2.17/1.95 eV for **3DPA‐DiKTa**, resulting in small Δ*E*_ST_ values of 0.21 eV for **3TPA‐DiKTa** and 0.22 eV for **3DPA‐DiKTa**. These values are almost identical to that of **DiKTa** (≈0.20 eV) and its derivatives.^[18]^ There is a notable small degree of positive solvatochromism observed for **3DPA‐DiKTa** (Figure S22), which is characteristic of MR‐TADF compounds. There is a more pronounced positive solvatochromism for **3TPA‐DiKTa**, consistent with a more significant admixture of CT character to the SRCT emissive excited state.

**Figure 5:**
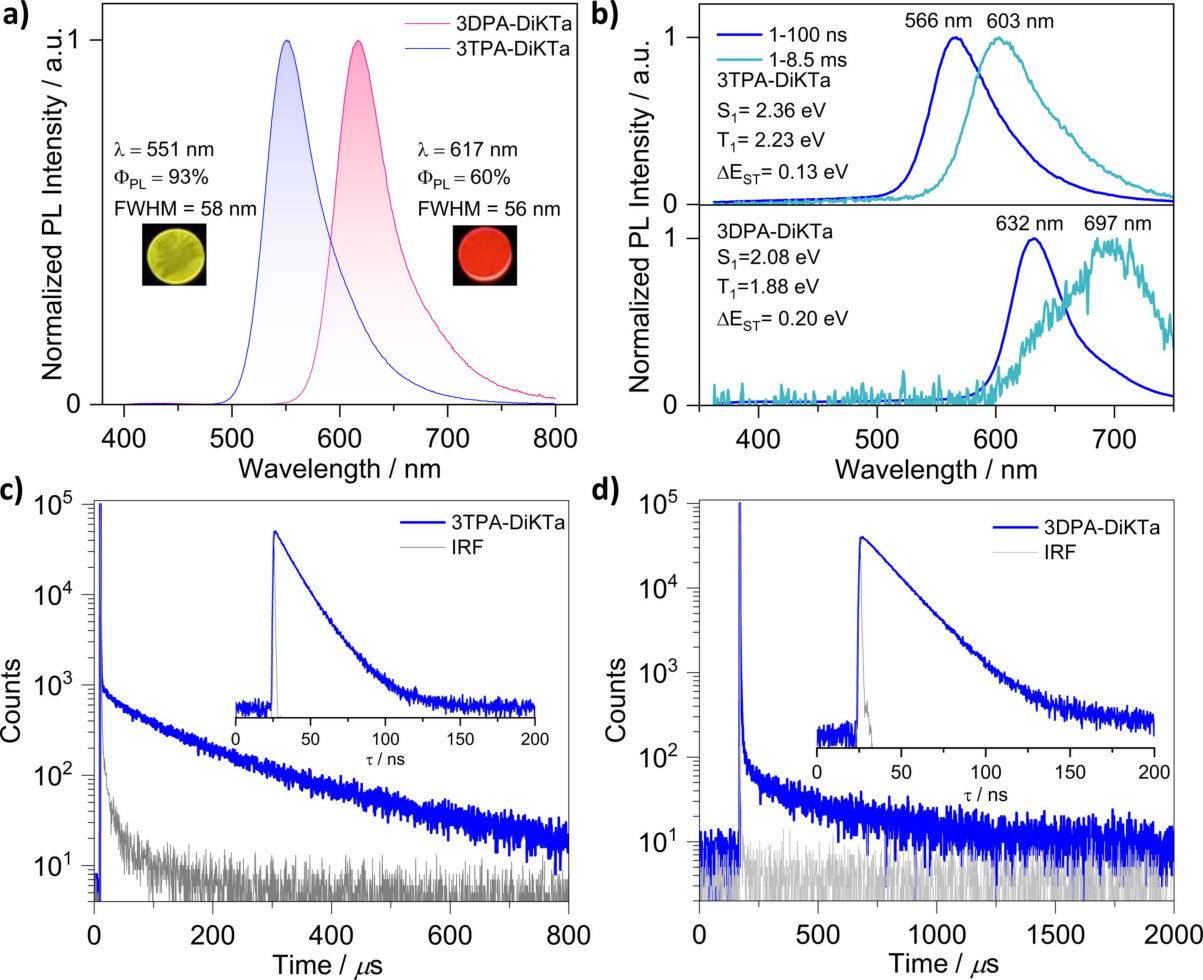
Photophysical properties of **3TPA‐DiKTa** and **3DPA‐DiKTa** in 2 wt % doped mCP films at room temperature. a) PL spectra (λ_exc_=340 nm); b) prompt PL and phosphorescence spectra measurement (λ_exc_=343 nm); c) and d) time‐resolved PL decays (λ_exc_=379 nm). The inset are photographs of the photoluminescence from the films.

We next investigated the photophysical behavior of both emitters in the OLED relevant host, mCP, which has a suitably high triplet energy of 2.91 eV.^[22]^ First, we identified 2 wt % as the optimal concentration by measuring the Φ_PL_ of spin‐coated films of varying concentration from 1 wt % to 10 wt % in mCP. We then compared the Φ_PL_ of 2 wt % films in a range of hosts such as bis[2‐(diphenylphosphino)phenyl]ether oxide (DPEPO) and 4,4′‐bis(9‐carbazolyl)‐1,1′‐biphenyl (CBP), and the Φ_PL_ in mCP was found to be the highest at 86 %. (Figure S23). The Φ_PL_ decreased along with a red‐shifted emission (Figure S24 and Table S6) upon an increase of the doping concentration of each emitter in mCP host. The red‐shifted emission can be ascribed in part to aggregate formation. As shown in Figure 5a, **3TPA‐DiKTa** emits at λ_PL_=551 nm with FWHM of 58 nm (245 meV) in 2 wt % mCP evaporated film, whereas **3DPA‐DiKTa** shows a more red‐shifted emission at λ_PL_=617 nm with FWHM of 56 nm (198 meV). Notably, their emission spectra in mCP are red‐shifted compared to those in dilute toluene, which given the low polarity of this host, implies the presence of host/guest interactions and likely contribution from aggregates in the solid state even at the low doping concentration employed.^[23]^ Benefiting from the delicate balance between CT and SRCT, **3TPA‐DiKTa** shows a higher Φ_PL_ of 93 % than **3DPA‐DiKTa** (Φ_PL_=60 %) in 2 wt % mCP evaporated film. The time‐resolved PL decays in mCP show a nanosecond prompt emission and a microsecond long delayed emission at room temperature (Table 1). **3TPA‐DiKTA** possesses a shorter delayed lifetime, τ_DF_, of 131 μs in comparison to **3DPA‐DiKTa** (τ_DF_ of 323 μs) in 2 wt % mCP doped films (Figures 5c–d). The temperature‐dependent time‐resolved PL decays are shown in Figure S25. The prompt emission is insensitive to temperature while the delayed emission is thermally activated, the latter behavior consistent with TADF. Based on the quantum yield and lifetime of prompt and delayed fluorescence, the kinetic parameters of the two emitters were calculated according to the methodology described in our previous report (Table S7).^[24]^ **3TPA‐DiKTa** possesses a slower singlet radiation decay (*k*_r_^s^) of 1.98×10^7^ s^−1^ compared to that of **3DPA‐DiKTa** (*k*_r_^s^=3.06×10^7^ s^−1^); these values are comparable to most of the reported MR‐TADF materials.^[25]^ **3DPA‐DiKTa** and **3TPA‐DiKTa** possess slow reverse intersystem crossing rate constants (*k*_RISC_) of 0.14 and 2.49×10^4^ s^−1^, respectively, values that are not uncommon for MR‐TADF emitters.^[26]^ The S_1_/T_1_ energies were determined from the onsets of the prompt fluorescence and phosphorescence spectra of 2 wt % mCP doped films of **3TPA‐DiKTa** and **3DPA‐DiKTa** at 77 K (Table 1). The Δ*E*_ST_ value is 0.13 eV for **3TPA‐DiKTa** and 0.20 eV for **3DPA‐DiKTa** (Figure 5b). The former is smaller than that of **DiKTa** (0.20 eV) and accompanied by a shorter τ_DF_, which is desirable for a TADF material.

**Table 1:** Photophysical data in 2 wt % doped mCP films.

Compound	λ_PL_^[a]^ [nm]	FWHM^[b]^ [nm]	Φ_PL_^[c]^ [%]	τ_p_^[d]^; τ_d_^[d]^ [ns; μs]	T_1_^[e]^ [eV]	S_1_^[f]^ [eV]	Δ*E*_ST_^[g]^ [eV]
**DiKTa**	466	40	70	4.5, 168	2.55	2.75	0.20
**3TPA‐DiKTa**	551	58	93	14, 131	2.23	2.36	0.13
**3DPA‐DiKTa**	617	56	60	16, 323	1.88	2.08	0.20

[a] Obtained at 300 K, λ_exc_=340 nm. [b] Full‐width at half‐maximum. [c] Photoluminescence quantum yield of thermally evaporated thin films, measured using an integrating sphere, under N_2_ at λ_exc_=340 nm. [d] Measured at λ_exc_=379 nm and 300 K under vacuum. [e] Obtained from the onset of the prompt spectrum (1–100 ns) at 77 K. [f] Obtained from the onset of the delayed spectrum (1–8.5 ms) at 77 K (λ_exc_=343 nm). [g] Δ*E*_ST_=*E*(S_1_)−*E*(T_1_).

**Figure 6:**
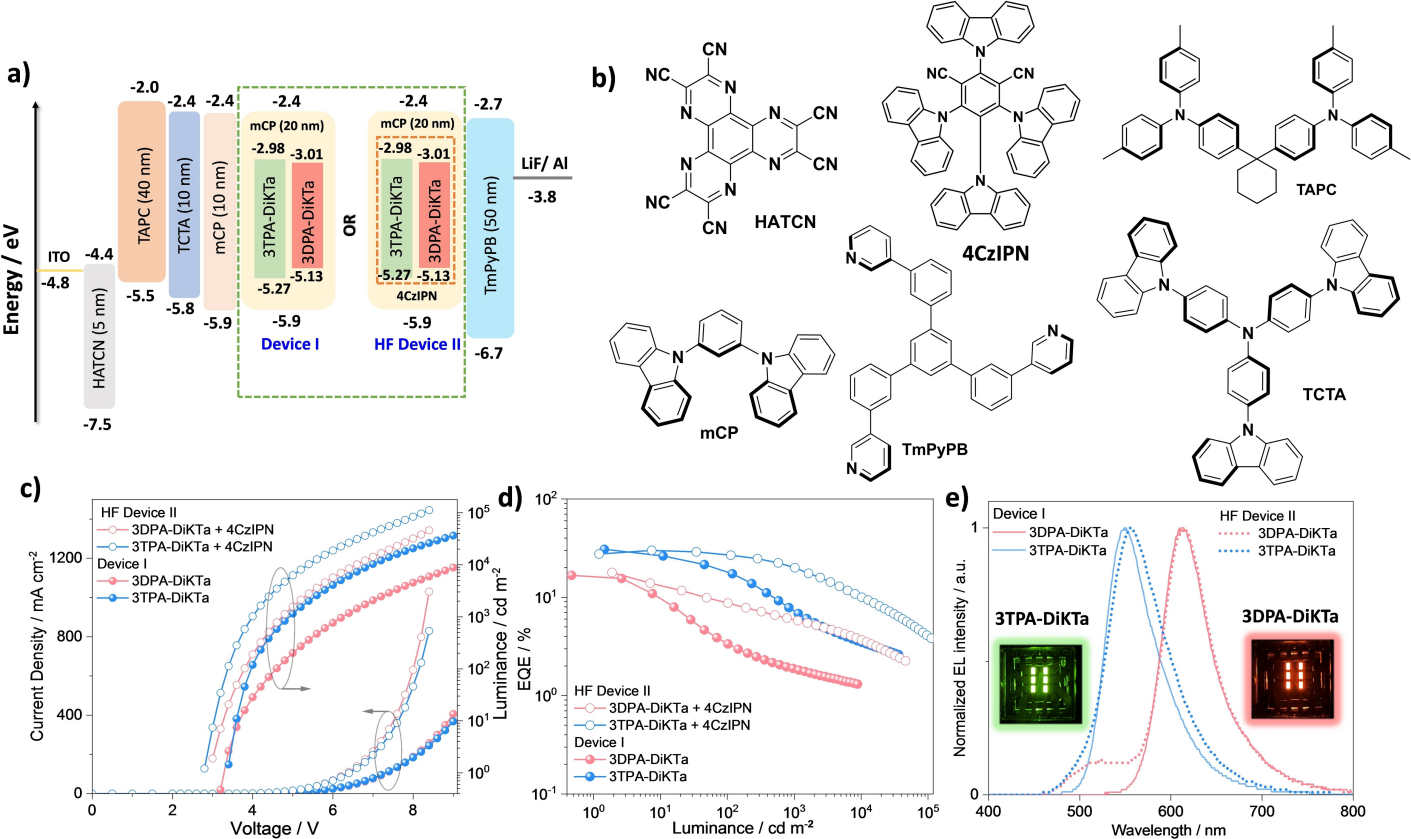
a) Energy level diagram of materials employed in the devices, the red dotted box in HF device **II** indicates the energy levels of the assistant dopant 4CzIPN. b) Molecular structure of materials used in the devices. c) Current density and luminance versus voltage characteristics for the devices. d) External quantum efficiency versus luminance curves for the devices. e) Electroluminescence spectra of the devices, the inset is photograph images of the electroluminescence from the devices.

Based on the promising optoelectronic properties, we next proceeded to fabricate vacuum‐deposited OLEDs using **3TPA‐DiKTa** and **3DPA‐DiKTa** as emitters. As shown in Figure 6a and Figure S34, the optimized OLED stack (device **I**) consisted of: indium‐tin‐oxide (ITO, 112 nm)/1,4,5,8,9,11‐hexaazatriphenylenehexacarbonitrile (HATCN) (5 nm)/1,1‐bis[(di‐4‐tolylamino)phenyl]cyclohexane (TAPC) (40 nm)/tris(4‐carbazoyl‐9‐ylphenyl)amine (TCTA) (10 nm)/1,3‐bis(*N*‐carbazolyl)benzene (mCP) (10 nm)/emissive layer (20 nm)/1,3,5‐tri[(3‐pyridyl)‐phen‐3‐yl]benzene (TmPyPB) (50 nm) LiF (0.6 nm)/Al (100 nm), where HATCN is the hole injection layer (HIL), TAPC and TCTA play the role of hole transporting layers (HTL) and mCP acts as an electron/exciton blocking layer (EBL). TmPyPB acts both as an electron transport layer and a hole blocking layer due to its deep HOMO (−6.7 eV),^[19]^ and LiF acts as an electron injection layer by modifying the work function of the aluminium cathode. The molecular structures of the materials used in these devices are shown in Figure 6b. The emission layer contained either 2 wt % of **3TPA‐DiKTa** or **3DPA‐DiKTa** doped into mCP host.

**Figure 7:**
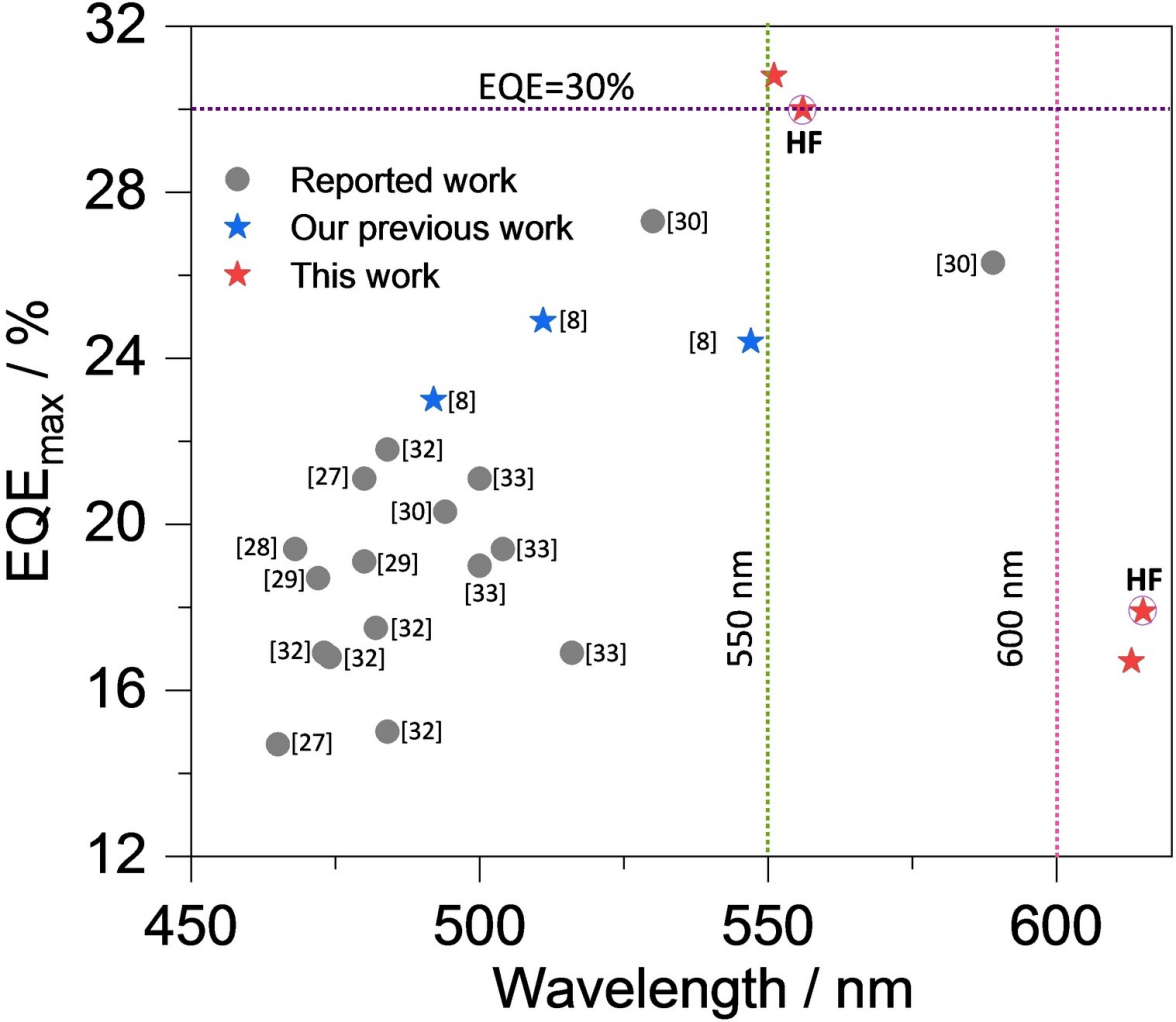
EQE_max_ of reported ketone‐based MR‐TADF OLEDs as a function of λ_EL_ (all the references in this Figure can be found in the Supporting Information).

The performance of the OLEDs is summarized in Table S2 and S8 and device fabrication statistics are provided in Figure S33. Current density—voltage—luminance (*J–V–L*) curves, EQE–luminance curves, and electroluminescence spectra (EL) are shown in Figures 6c–e. Both OLEDs show narrow electroluminescence with a FWHM of 62 nm (267 meV) for the device with **3TPA‐DiKTa** emitting at λ_EL_ of 551 nm and a FWHM is 60 nm (203 meV) for the device with **3DPA‐DiKTa** emitting at λ_EL_ of 613 nm (Figure 6e). The EL spectra are similar to their corresponding PL spectra in the mCP doped thin film (Figure S27) reflecting an emission from the same SRCT excited state. In comparison to the previously reported OLED with **DiKTa** (λ_EL_=465 nm, FWHM=39 nm), both of the devices showed a red‐shifted emission with a slightly broader electroluminescence.^[7]^ As shown in Table S9 and Figure 7, compared to reported OLED devices with derivatives of **DiKTa** bearing peripheral electron‐donating groups, the **3TPA‐DiKTa**‐based OLEDs showed red‐shifted emission while showing the best performance of green‐emitting ketone‐based MR‐TADF OLEDs. The **3DPA‐DiKTa**‐based OLEDs are one of the highest‐efficiency red‐emissive devices containing a ketone‐based MR‐TADF emitters (Figure 7).[^8^, ^13^] We note that, besides the DiKTa based red MR‐TADF OLEDs, there are only a small number of red MR‐TADF OLEDs. These include emitters based on **BCz** ^[10b]^ such as **BBCZ‐*tert*‐butyl** (**5**) (λ_EL_=615 nm, FWHM=26 nm, EQE_max_=22 %) and boron‐nitrogen embedded polycyclic heteroaromatics^[27]^ such as **R‐BN** (λ_EL_=664 nm, FWHM=48 nm, EQE_max_=28.1 %) and **R‐BNT** (λ_EL_=686 nm, FWHM=49 nm, EQE_max_=27.6 %). The corresponding Commission Internationale de l’Éclairage (CIE) coordinates are (0.409, 0.577) and (0.633, 0.365) for the devices with **3TPA‐DiKTa** and **3DPA‐DiKTa**, respectively. The **3TPA‐DiKTa** based device showed a much higher EQE_max_ in comparison to the device with **3DPA‐DiKTa** as well as other reported DiKTa based devices (Table S9). The average over the set of 23 pixels of **3TPA‐DiKTa** devices showed a EQE_max_ of 30.8 %, a maximum current efficiency (CE_max_) of 117 cd/A, and maximum power efficiency (PE_max_) of 108 lm W^−1^ at 1.45 cd m^−2^ (Table S2 and Figures S31–S32). Three of the 23 pixels showed recorded EQE_max_ close to 40 % at a luminance 1.84 cd m^−2^ (Figure S33). By contrast, the **3DPA‐DiKTa**‐based device showed an EQE_max_ of 16.7 %, CE_max_=28 cd A^−1^ and PE_max_=27 lm W^−1^, the brightness only reached 8800 cd m^−2^ at an EQE of 1.3 % (Table 2, Figures 6 and S31–32). However, although OLEDs with both emitters show very high efficiency, serious efficiency roll‐off was observed where the external quantum efficiency at 100 cd m^−2^ (EQE_100_) of the devices with **3TPA‐DiKTa** and **3DPA‐DiKTa** reached only 18.1 % and 3.4 %, respectively. The long delayed lifetime and slow RISC rate contribute to increased triplet‐triplet annihilation and triplet‐polaron annihilation, both of which lead to efficiency roll‐off at higher current densities in the device.^[28]^

**Table 2:** Electroluminescence data for the devices.

Device	λ_EL_^[c]^ [nm]	FWHM^[c]^ [nm]	CIE^[c]^ (x y)	V_on_^[d]^ [V]	CE_max_ [cd A^−1^]	PE_max_ [lm W^−1^]	*L*_max_^[e]^ [cd m^−2^]	EQE^[f]^ [%]
Device **I** ^[a]^								
**3TPA‐DiKTa**	551	62	0.409, 0.577	3.4	117	108	36 400	30.8/18.1/7.3
**3DPA‐DiKTa**	613	60	0.633, 0.365	3.2	28	27	8800	16.7/3.4/1.9

HF Device **II** ^*b*^								
**3TPA‐DiKTa**	556	70	0.424, 0.551	3.0	106	111	11 2190	30.0/27.4/20.0
**3DPA‐DiKTa**	615	61	0.585, 0.396	3.0	35	37	46 003	17.9/8.7/6.0

[a] Device **I**; ITO/HATCN (5 nm)/TAPC (40 nm)/TCTA (10 nm)/mCP (10 nm)/emissive layer (2 wt % emitter in mCP, 20 nm)/TmPyPB (50 nm)/LiF (0.6 nm)/Al (100 nm. [b] HF Device **II**; ITO/HATCN (5 nm)/TAPC (40 nm)/TCTA (10 nm)/mCP (10 nm)/emissive layer (10 wt % 4CzIPN and 2 wt % emitter in mCP, 20 nm)/TmPyPB (50 nm)/LiF (0.6 nm)/Al (100 nm). [c] The electroluminescence maximum, CIE coordinates and FWHM of the EL spectrum recorded at 5 V. [d] The turn‐on voltage at EQE_max_. [e] Luminance maximum *(L*_max_) measured at the highest voltage (9 V for device **I** and 8.4 V for device **II**). [f] The order of measured values: the EQE_max_/EQE_100_/EQE_1000_.

**Figure 8:**
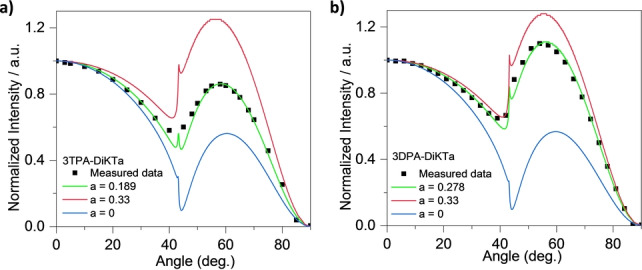
Measured PL intensity of the p‐polarized emission as a function of a rotation angle of mCP films doped with different emitters films at peak emission wavelength and comparison with simulated results: a) **3TPA‐DiKTa** and b) **3DPA‐DikTa**.

To overcome the severe efficiency roll‐off, TADF‐sensitized‐fluorescence HF devices (HF device **II**) were fabricated.^[29]^ Here, 4CzIPN was selected as the TADF assistant dopant because of its high Φ_PL_ ^[30]^ and short τ_d_ of 3.0 μs in mCP (Figure S30) as well as the strong spectra overlap between the absorption spectra of **3TPA‐DiKTa** and **3DPA‐DiKTa** in toluene and the PL spectrum of 10 wt % 4CzIPN in mCP (Figure S28). Based on a concentration screen (Figure S29), the optimized ratio of mCP/4CzIPN/emitters was determined to be 88 : 10 : 2. The delayed lifetime of the 2 wt % **3TPA‐DiKTa**:10 wt % 4CzIPN: 88 wt % mCP doped film is 3.3 μs, which is much shorter than that in doped film (131 μs) without the assist dopant. Based on this analysis, the HF device **II** contained an emissive layer consisting of 2 wt % **3TPA‐DiKTa** or 2 wt % **3DPA‐DiKTa**: 10 wt % 4CzIPN: 88 wt % mCP (Figure 6a). The device performances are shown in Figures 6 and S31–S32, and the data are summarized in Table 2. As shown in Figure 6e, the EL spectrum of the HF devices **II** with **3TPA‐DiKTa** and **3DPA‐DiKTa** are similar to the corresponding devices **I**. The HF devices **II** show an EQE_max_ of 30.0 % at λ_EL_=556 nm (FWHM=70 nm) with **3TPA‐DiKTa** as the terminal emitter, and an EQE_max_ of 17.9 % at λ_EL_=615 nm (FWHM=61 nm) with **3DPA‐DiKTa** as the terminal emitter, performances that are comparable to the analogous devices **I** (Table 2 and Figure 6). More significantly, the efficiency roll‐off was much improved for both HF devices **II** (Figure 6d and Table 2). The EQEs at 100 and 1000 cd m^−2^ in the HF devices **II** with **3TPA‐DiKTa** are 27.4 % and 20.0 %, and with **3DPA‐DiKTa** are 8.7 % and 6.0 %, respectively (Table 2). This corresponds to a significantly lower efficiency roll‐off from 41 % to 8.6 % for devices with **3TPA‐DiKTa** and from 80 % to 51 % for devices with **3DPA‐DiKTa** at 100 cd m^−2^, respectively. assuming 25 % out coupling efficiency, the EQE_max_ of the **3TPA‐DiKTa** device is expected to be 23.3 %, which is much lower than the observed EQE_max_ of 30.8 % whilst the calculated EQE_max_ of the **3DPA‐DiKTa** device is 15.0 %, and very close to the observed EQE_max_ of 16.7 %. One potential explanation of the much‐improved **3TPA‐DiKTa** OLED efficiency would be that the transition dipole moment (TDM) of the emitter is preferentially horizontally oriented, parallel to the substrate surface.^[31]^ We therefore measured the orientation of the TDM of the emitters in 2 wt % evaporated doped films in mCP of 50 nm thickness, emulating the thickness in the device. The angular dependent PL measurement results are shown in Figure 8 and used refractive index measurements and modelling are shown in Figure S1. The anisotropy factors, *a*, which is defined by the ratio of emitted power by vertical dipoles to total emitted power by all dipoles and extracted from the p‐polarized emission, were found to be 0.189 for **3TPA‐DiKTa**, and 0.278 for **3DPA‐DiKTa**; notably, previously reported DiKTa derivatives showed anisotropy factors close to isotropic at 0.33.^[8]^ The relatively horizontal transition dipole moment orientation in **3TPA‐DiKTa** is due to its high molecular weight and higher degree of planarity in contrast to **3DPA‐DiKTa** (based on the DFT calculated optimized structure in the gas phase).^[31]^ Considering the device structure and the measured anisotropic factors, the corresponding simulated out‐coupling efficiency calculated for **3TPA‐DiKTa** is 28.3 % and for **3DPA‐DiKTa** is 20.5 %. Combining the measured Φ_PL_ of the films and the simulated outcoupling efficiencies, the calculated EQE_max_ values are 26.4 % for **3TPA‐DiKTa** and 12.3 % for **3DPA‐DiKTa**, which are slightly lower than the observed values. Therefore, the emitter orientation alone cannot explain this inconsistency. Similar higher than expected efficiencies of OLEDs have also been reported in many other MR‐TADF device studies.^[8]^ Although the origin of the higher‐than‐expected EQE_max_ is not clear here, we can envision that a potential cause is the Purcell effect associated with the reflective electrodes in the OLED stack, which can result in a higher than expected IQE.^[32]^ A second potential explanation could be microcavity effects in the OLED stack leading to light emission that is directed forwards more than for a Lambertian emitter and hence increasing the apparent EQE when measured from the forward direction.^[8]^

## Conclusions

This study reports green and red‐emitting MR‐TADF compounds based on a DiKTa core. **3TPA‐DiKTa** and **3DPA‐DiKTa** show high Φ_PL_, moderate Δ*E*_ST_ values of 0.13 and 0.20 eV, and long delayed lifetimes of 131 and 323 μs in 2 wt % doped mCP films, respectively. OLEDs using these MR‐TADF materials showed excellent performance with record‐high EQE_max_ of 30.8 % for ketone‐based MR‐TADF OLEDs in the case of the green‐emitting device with **3TPA‐DiKTa** (λ_EL_ of 551 nm) and of 16.7 % for the red‐emitting device with **3DPA‐DiKTa** (λ_EL_ of 613 nm). Efficiency roll‐off could be mitigated through the use of a hyperfluorescence device structure using 4CzIPN as an assistant dopant. An important contribution to the outstanding EQE of the **3TPA‐DiKTa** is horizontal alignment of the transition dipole moment of the emitter in the evaporated film. These results demonstrate that simple decoration of the DiKTa acceptor with a TPA and DPA substituent is an effective approach to attaining efficient green/red TADF OLEDs.

## Supporting Information

^1^H and ^13^C NMR spectra, HRMS and HPLC of all target compounds; supplementary computational data; supplementary photophysical data, supplementary devices data, and orientation measurement data.

## Conflict of interest

The authors declare no conflict of interest.

1

## Supporting information

As a service to our authors and readers, this journal provides supporting information supplied by the authors. Such materials are peer reviewed and may be re‐organized for online delivery, but are not copy‐edited or typeset. Technical support issues arising from supporting information (other than missing files) should be addressed to the authors.

Supporting Information

Supporting Information

## Data Availability

The research data supporting this publication can be accessed at https://doi.org/10.17630/c6d59db2‐3168‐4152‐a520‐fe0d7012cca7
