# Supplementary material for: Highly Efficient Green and Red Narrowband Emissive Organic Light‐Emitting Diodes Employing Multi‐Resonant Thermally Activated Delayed Fluorescence Emitters
Source: Angew Chem Int Ed Engl. 2022 Nov 23;61(52):e202213697. doi: 10.1002/anie.202213697 (PMC10100094; doi:10.1002/anie.202213697)
Supplement: Supplementary file 1 — Supporting Information [file ANIE-61-0-s002.pdf]

Angewandte Chemie
Eine Zeitschrift der Gesellschaft Deutscher Chemiker

Supporting Information

Highly Efficient Green and Red Narrowband Emissive Organic Light-Emitting Diodes Employing Multi-Resonant Thermally Activated Delayed Fluorescence Emitters

S. Wu, A. Kumar Gupta, K. Yoshida, J. Gong, D. Hall, D. B. Cordes, A. M. Z. Slawin, I. D. W. Samuel, E. Zysman-Colman**

Supporting Information

Highly Efficient Green & Red Narrowband Emissive Organic Light-Emitting Diodes Employing Multi-Resonant Thermally Activated Delayed Fluorescence Emitters

Sen Wu,[a†] *Abhishek Kumar Gupta,*[a,b†] *Kou Yoshida,*[b] *Junyi Gong,*[b] *David Hall,*[a] *David B. Cordes,*[a] *Alexandra M. Z. Slawin,*[a] *Ifor D. W. Samuel*[b*] *and Eli Zysman-Colman*[a*]

[a]Organic Semiconductor Centre, EaStCHEM School of Chemistry, University of St Andrews, St Andrews, Fife, UK, KY16 9ST, Fax: +44-1334 463808; Tel: +44-1334 463826; E-mail: eli.zysman-colman@st-andrews.ac.uk.

[b]Organic Semiconductor Centre, SUPA School of Physics and Astronomy, University of St Andrews, St Andrews, UK, KY16 9SS; E-mail: idws@st-andrews.ac.uk

Table of Contents

General methods **S3**

Literature Study **S11**

X-Ray structural analysis **S12**

Calculation **S14**

Synthesis **S19**

Optoelectronic characterization **S27**

OLEDs fabrication and characterization **S31**

Reference **S36**

General methods

General Synthetic Procedures. All reagents and solvents were obtained from commercial sources and used as received unless otherwise stated. Air-sensitive reactions were done under a nitrogen atmosphere using Schlenk techniques. Dry solvents used in the reaction were obtained from a MBRAUN SPS5 solvent purification system. Flash column chromatography was carried out using silica gel (Silia-P from Silicycle, 60 Å, 40-63 µm). Analytical thin-layer-chromatography (TLC) was performed with silica plates with aluminium backings (250 µm with F-254 indicator). TLC visualization was accomplished by 254/365 nm UV lamp. HPLC analysis was conducted on a Shimadzu LC-40 HPLC system. HPLC traces were performed using a Shim-pack GIST 3µm C18 reverse phase analytical column. ^{1}H and ^{13}C and NMR spectra were recorded on a Bruker Advance spectrometer (500 MHz for ^{1}H and 126 MHz for ^{13}C). The following abbreviations have been used for multiplicity assignments: "s" for singlet, "d" for doublet, "t" for triplet, "m" for multiplet, "dd" for doublet of doublets, "dt" for doublet of triplets. ^{1}H and ^{13}C NMR spectra were referenced to the solvent peaks). Melting points were measured using open-ended capillaries on an Electrothermal 1101D Mel-Temp apparatus and are uncorrected. High-resolution mass spectrometry (HRMS) was performed at University of Edinburgh Mass Spectrometry Facility. Elemental analyses were performed by Dr. Joe Casillo at the University of Edinburgh.

Electrochemistry measurements. Cyclic Voltammetry (CV) analysis was performed on an Electrochemical Analyzer potentiostat model 620E from CH Instruments at a sweep rate of 100 mV/s. Differential pulse voltammetry (DPV) was conducted with an increment potential of 0.004 V and a pulse amplitude, width, and period of 50 mV, 0.05, and 0.5 s, respectively. Samples were prepared in DCM solutions, which were degassed

by sparging with DCM-saturated nitrogen gas for 5 minutes prior to measurements. All measurements were performed using 0.1 M DCM solution of tetra-*n*-butylammonium hexafluorophosphate, [nBu$_4$N]PF$_6$]. An Ag/Ag$^+$ electrode was used as the reference electrode while a platinum electrode and a platinum wire were used as the working electrode and counter electrode, respectively. The redox potentials are reported relative to a saturated calomel electrode (SCE) with a ferrocenium/ferrocene (Fc/Fc$^+$) redox couple as the internal standard (0.46V vs SCE).[1]

Photophysical measurements. Optically dilute solutions of concentrations on the order of 10^{-5} or 10^{-6} M were prepared in spectroscopic or HPLC grade solvents for absorption and emission analysis. Absorption spectra were recorded at room temperature on a Shimadzu UV-2600 double beam spectrophotometer with a 1 cm quartz cuvette. Molar absorptivity determination was verified by linear regression analysis of values obtained from at least four independent solutions at varying concentrations with absorbance ranging from 0.025 to 0.100.

For emission studies, steady-state emission, excitation spectra and time-resolved emission spectra were recorded at room temperature using an Edinburgh Instruments FLS980 fluorimeter. Samples were excited at 340 nm for steady-state measurements. Photoluminescence quantum yields for solutions were determined using the optically dilute method,[2] in which four sample solutions with absorbances of ca. 0.10, 0.075, 0.050 and 0.025 at 342 nm were used. The Beer-Lambert law was found to remain linear at the concentrations of the solutions. For each sample, linearity between absorption and emission intensity was verified through linear regression analysis with the Pearson regression factor (R^2) for the linear fit of the data set surpassing 0.9. Individual relative quantum yield values were calculated for each solution and the values reported represent the slope obtained from the linear fit of these results. The

quantum yield of the sample, Φ_{PL}, can be determined by the equation $\Phi_{PL} = (\Phi_r * \frac{A_r}{A_s} * \frac{I_s}{I_r} * \frac{n_s^2}{n_r^2})$,[2] where A stands for the absorbance at the excitation wavelength (λ_{exc} = 342 nm), I is the integrated area under the corrected emission curve and n is the refractive index of the solvent with the subscripts "s" representing sample and "r" representing reference. Φ_r is the absolute quantum yield of the external reference Rhodamine 6G (Φ_r = 95% in ethanol).[3]

An integrating sphere (Hamamatsu, C9920-02) was employed for the photoluminescence quantum yield measurements of thin film samples.[4] The Φ_{PL} of the films were then measured in air and N_2 environment by purging the integrating sphere with N_2 gas flow. The photophysical properties of the film samples were measured using an Edinburgh Instruments FS980 fluorimeter. Time-resolved PL measurements of the thin films were carried out using the multi-channel scaling (MCS) technique. The samples were excited at 379 nm by a pulsed laser diode (PicoQuant, LDH-D-C-375, FWHM < 40 ps, pulse energy = 58.5 ± 1.2 pJ, peak power = 1.5 ± 0.3 W, laser spot diameter = 0.4 ± 0.1 mm, power density = 11.6 ± 3.7 mW/cm^2) and were kept in a vacuum of < 8 × 10^{-4} mbar. The singlet and triplet state energies were determined from the onset values of the prompt fluorescence and phosphorescence spectra at 77 K. The singlet-triplet energy gap (ΔE_{ST}) was estimated from the difference in energy of the prompt fluorescence and phosphorescence spectra. Phosphorescence spectra were measured from 1 ms after photoexcitation with an iCCD exposure time of 8.5 ms. Prompt fluorescence spectra were measured from 1 ns after photoexcitation with an iCCD exposure time of 100 ns. The films were excited by a femtosecond laser emitting at 343 nm (Orpheus-N, model: SP-06-200-PP). Emission from the samples was focused onto a spectrograph (Chromex imaging, 250is spectrograph) and detected on a sensitive

gated iCCD camera (Stanford Computer Optics, 4Picos) having sub-nanosecond resolution.

Fitting of time-resolved luminescence measurements: Time-resolved PL measurements were fitted to a sum of exponentials decay model, with chi-squared (χ^2) values between 1 and 2, using the EI FLS980 Each component of the decay is assigned with a weight, (w_i), which is the contribution of the emission from each component to the total emission.

The average lifetime was then calculated using the following expressions:[5]

1. Two exponential decay model:

$$\tau_{AVG} = \tau_1 w_1 + \tau_2 w_2 \qquad \text{(S1)}$$

with weights defined as $w_1 = \frac{A1\tau_1}{A1\tau_1 + A2\tau_2}$ and $w_2 = \frac{A2\tau_2}{A1\tau_1 + A2\tau_2}$ where A_1 and A_2 are the preexponential-factors of each component.

2. Three exponential decay model:

$$\tau_{AVG} = \tau_1 w_1 + \tau_2 w_2 + \tau_3 w_3 \qquad \text{(S2)}$$

with weights defined as $w_1 = \frac{A1\tau_1}{A1\tau_1 + A2\tau_2 + A3\tau_3}$, $w_2 = \frac{A2\tau_2}{A1\tau_1 + A2\tau_2 + A3\tau_3}$ and $w_3 = \frac{A3\tau_3}{A1\tau_1 + A2\tau_2 + A3\tau_3}$ where A_1, A_2 and A_3 are the preexponential-factors of each component.

OLED Fabrication and Characterization: The OLED devices were fabricated in a bottom-emitting structure via thermal evaporation in a high vacuum at a base pressure of <5×10^{-7} mbar. A pre-patterned glass substrate coated with indium doped tin oxide (ITO) was cleaned sequentially by ultrasonication in acetone, and isopropanol for 15

minutes. The temperature of ultrasonication bath was set at 60-70 °C. The cleaned substrate was exposed to oxygen plasma for 3 min to remove all dust and organics on the ITO surface and to increase the work function of ITO anode for better hole injection from the anode to organic layer. The substrate was loaded in the thermal evaporator. Organic layers were deposited at a rate of 0.3-1.0 Å/s, monitored using a quartz crystal. The electron injection layer, LiF, was deposited at a rate of 0.05 Å/s, while the Al cathode was deposited initially with a rate of 0.5 Å/s to obtain 10 nm thickness and after that the rate of Al cathode was increased to 3 Å/s. Two custom-made shadow masks were used to define the area of the evaporations. The organic layers and LiF were evaporated with a same shadow mask but Al was evaporated with the other mask. The active area of the OLED was 2 mm^2, determined by the spatial overlap of the anode and cathode electrodes. All the devices were encapsulated with glass lids and UV epoxy resin inside a N_2 filled globe box. The luminance-current-voltage characteristics were measured in an ambient environment using a Keithley 2400 source meter and a homemade photodiode circuit connected to a Keithley 2000 multimeter for the voltage reading. The external quantum efficiency was calculated assuming Lambertian emission pattern for the OLEDs. The electroluminescence spectra were recorded by an Andor DV420-BV CCD spectrometer.

Determination of emitter dipole orientation by angle-resolved PL measurement:

Dipole orientation of emitter molecules was determined by angle-resolved PL measurements of thin films doped with each emitter.[6] The doping concertation of the films are same as the ones used for the OLEDs, i.e., 2 wt%, while the thicknesses of these films are around 50 nm. To quantify this, an anisotropy factor (a) was used, which is defined by the ratio of emitted power by vertical dipoles to total emitted power by all dipoles.[7] We note that for perfectly horizontal dipole orientation (i.e. parallel to the

substrate surface), a = 0, for isotropic orientation, a = 1/3, and for perfectly vertical orientation, a = 1. The details of our set-up and calculation can be found in reference.[8] We obtained the optical constants and thickness of each organic layer using a variable angle spectroscopic ellipsometer (J. A. Woollam, M-2000 Ellipsometer) to measure films deposited on glass substrates.The fitting process to obtain optical constants is that staring with the Cauchy model for non-absorbing region, where no absorption of dopants is found, we fitted absorbing region with b-spline model. The fitting to the non-absorbing region of **3TPA-DiKTa** film is from 570 nm and the one of **3DPA-DiKTa** film is from 650 nm. Figure **S1** shows the refractive index spectra of each the organic film. The observed refractive index spectra of mCP neat film and doped film of both molecules in mCP are quite similar. The small shoulders at 380 nm of the spectrum of the **3TPA-DiKTa** film originates from the absorption by **3TPA-DiKTa**, which was confirmed by comparison with the absorption spectrum of the dopant in toluene solution.

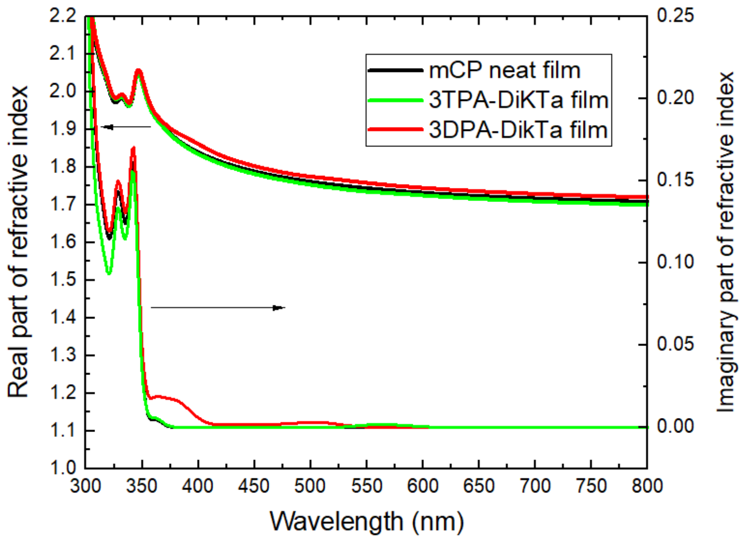

Figure **S1.** Refractive index spectra of each organic layer fabricated on glass substrate.

Calculation of out-coupling efficiency of OLEDs: The out-coupling simulation of the OLEDs is based on emission dipole as forced damped harmonic oscillator and

embedded in thin film stacks.[9] The details of the calculation can be found in reference.[8]

In the optical calculation, it was assumed that emitter dipole is localized at the mCP and TmPyPB interface. This is reasonable because the hole conduction is dominant in the host, mCP. The actual dipole position within the emission layer was not determined. Errors of 10% in maximum for the **3TPA-DiKTa** and around 30% for **3DPA-DiKTa** were estimated (See Figure **S2**).

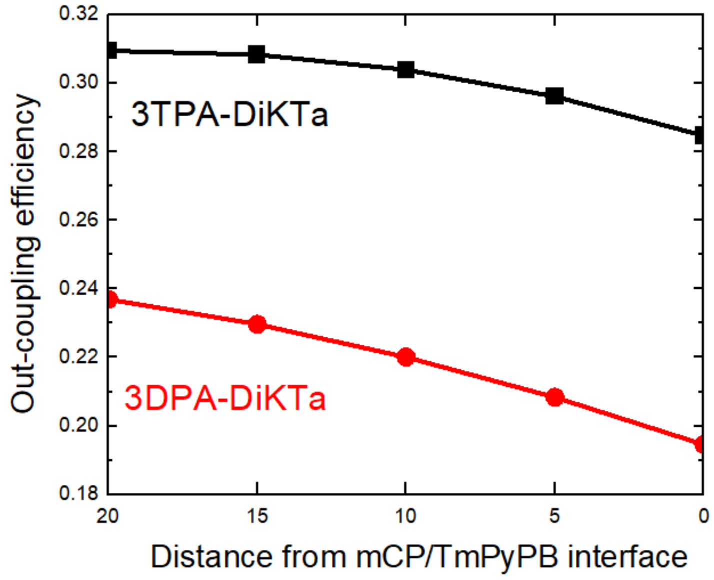

Figure **S2**. Effect of dipole position on the out-coupling efficiency of the OLEDs with different **3TPA-DiKTa** and **3DPA-DiKTa**. Dipole position was changed within the emission layer and plotted as a function of distance from mCP/TmPyPB interface.

Quantum chemical calculations. The calculations were performed using Density Functional Theory (DFT) within Gaussian 16[10] as well as the second order algebraic diagrammatic construction Spin-Component Scaling (ADC (2)-SCS) method using the Turbomole/7.5 package. For the DFT calculation, the ground state was optimized with PBE0[11] functional and the 6-31G(d,p) basis set,[12] and excited state calculations have been performed using Time-Dependent DFT within the Tamm-Dancoff approximation

(TDA-DFT)[13] with the same functional and basis set as for the ground state geometry optimization in gas phase. The molecular orbitals were visualized with Gaussview 5.0 software. For the ADC(2) calculation, the ground states was optimized with ADC (2)-SCS method and cc-pVDZ basis set in gas phase based on the geometry calculated by DFT.[14] Vertical excited states were performed on the ground state optimized structure using ADC(2)-SCS method. Different density plots were used to visualize change in electronic density between the ground and excited state and were visualized using the VESTA package.[15]

Literature Study

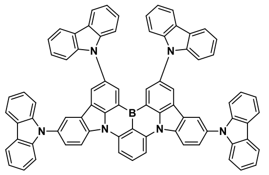

TCz-B

λ_{PL}=517 nm, Φ_{PL}= 89% in 1 wt% mCBP film
EQE_{max}= 29.2%

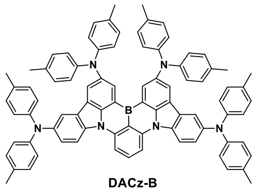

DACz-B

λ_{PL}=576 nm, Φ_{PL}= 87% in 1 wt% mCBP film
EQE_{max}= 19.6%

Angew. Chem. Int. Ed. **2021**, 60, 23142

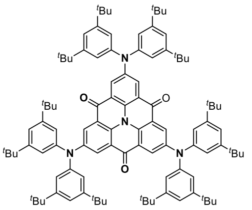

mBDPA-TOAT

λ_{PL}=599 nm, Φ_{PL}= 73% in 3 wt% mCBP film
EQE_{max}= 17.3%

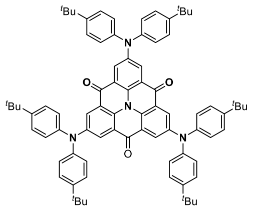

pBDPA-TOAT

λ_{PL}=603 nm, Φ_{PL}= 57% in 3 wt% mCBP film
EQE_{max}= 11.3%

Adv. Optical Mater. **2021**, 2101789

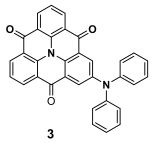

3

Φ_{PL}= 50% in 3 wt% mCBP film
EQE_{max}= 10%, CIE (0.46,0.54)

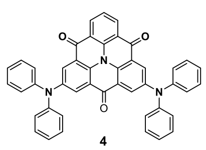

4

Φ_{PL}= 46% in 3 wt% mCBP film
EQE_{max}= 1% ,CIE (0.41,0.56)

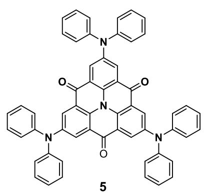

5

Φ_{PL}= 46% in 3 wt% mCBP film
EQE_{max}= 2%, CIE (0.57,0.43)

Adv. Optical Mater. **2021**, 2002174

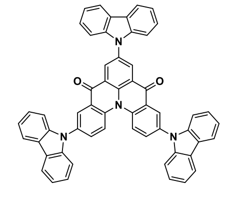

3Cz-DiKTa

λ_{PL}=539 nm, Φ_{PL}= 78% in 2 wt% mCBP film
EQE_{max}= 24.4%
ACS Appl. Mater. Interfaces, **2022,** 14, 19, 22341

Figure **S3**. Molecular structures discussed in the introduction.

X-Ray structure analysis

X-ray diffraction data for **3TPA-DiKTa** and **3DPA-DiKTa** were collected at 173 K using a Rigaku MM-007HF High Brilliance RA generator/confocal optics with XtaLAB P100 or P200 diffractometer [Cu Kα radiation (λ = 1.54187 Å)]. Intensity data were collected using either both ω and φ steps or solely ω steps, accumulating area detector images spanning at least a hemisphere of reciprocal space. Data for both compounds were collected using CrystalClear[16] and processed (including correction for Lorentz, polarization and absorption) using CrysAlisPro. [17] Structures were solved by dual-space methods (SHELXT)[18] and refined by full-matrix least-squares against F^2 (SHELXL-2018/3)[19]. Non-hydrogen atoms were refined anisotropically, and hydrogen atoms were refined using a riding model. All crystals of **3TPA-DiKTa** showed very weak diffraction at higher angles, even with long exposures, often showing no diffraction above 1.10 Å. This likely arises from a combination of regions of diffuse solvent in the structure as well as the extent of disorder present. Despite the weak diffraction from this compound, the structure could still be unambiguously determined. Both structures showed some disorder in peripheral phenyl rings, this was extensive in **3TPA-DiKTa**, and extended to one of the phenylene bridges. Disorder modelling included restraints to distances, angles and thermal motion as needed, and several of the disordered peripheral phenyl rings were constrained to an idealised geometry. Thermal ellipsoids in the DiKTa core of **3TPA-DiKTa** suggested that the core might be somewhat disordered as well, however this could not be successfully modelled. Both structures showed regions of void space containing diffuse electron density (**3TPA-DiKTa**: 218 Å^3, **3DPA-DiKTa**: 283 Å^3) and the SQUEEZE[20] routine implemented in PLATON[21] was used to remove the contribution to the diffraction pattern of the unordered electron density in the void spaces. Despite treatment with

SQUEEZE, the structure of **3TPA-DiKTa** showed higher than anticipated values of R_1 and wR_2, likely due to the extent of the disorder and inability to successfully model disorder into the DiKTa core of the molecule. All calculations except SQUEEZE were performed using the Olex2 interface.[22] Selected crystallographic data are presented in Table S1. Deposition numbers 2183420 and 2183421 contain the supplementary crystallographic data for this paper. These data are provided free of charge by the joint Cambridge Crystallographic Data Centre and Fachinformationszentrum Karlsruhe Access Structures service www.ccdc.cam.ac.uk/structures.

Table S1. Selected crystallographic data for **3TPA-DiKTa** and **3DPA-DiKTa**.

	**3TPA-DiKTa**	**3DPA-DiKTa**
Empirical formula	$C_{74}H_{50}N_4O_2$	$C_{57}H_{40}Cl_2N_4O_2$
Formula mass	1027.18	883.83
Colour, habit	Red prism	Red plate
Crystal size (mm^3)	0.28×0.06×0.04	0.15×0.04×0.01
Crystal system	Triclinic	Triclinic
Space group	$P\bar{1}$	$P\bar{1}$
a (Å)	10.9144(11)	11.0801(2)
b (Å)	17.2054(11)	12.5559(2)
c (Å)	17.3910(12)	17.2089(3)
α (°)	66.543(6)	88.8930(10)
β (°)	80.988(7)	89.4880(10)
γ (°)	76.734(7)	83.6380(10)
Volume/Å^3	2908.2(4)	2378.87(7)
Z	2	2
ρ_{calc}/g $^{cm-3}$	1.173	1.234
μ/mm^{-1}	0.549	1.592
F(000)	1076	920
Reflections collected	30332	28720
Independent reflections (R_{int})	10312 (0.0713)	9419 (0.0215)
Parameters, restraints	860, 1360	641, 225
Goodness-of-fit on F^2	1.504	1.058
R_1 [$I > 2\sigma(I)$]	0.1756	0.0462
R_1 (all data)	0.2497	0.0527
wR_2 [$I > 2\sigma(I)$]	0.4654	0.1309
wR_2 (all data)	0.5199	0.1384
Largest diff. peak/hole / e Å^{-3}	1.055, -0.361	0.300, -0.51

Calculations

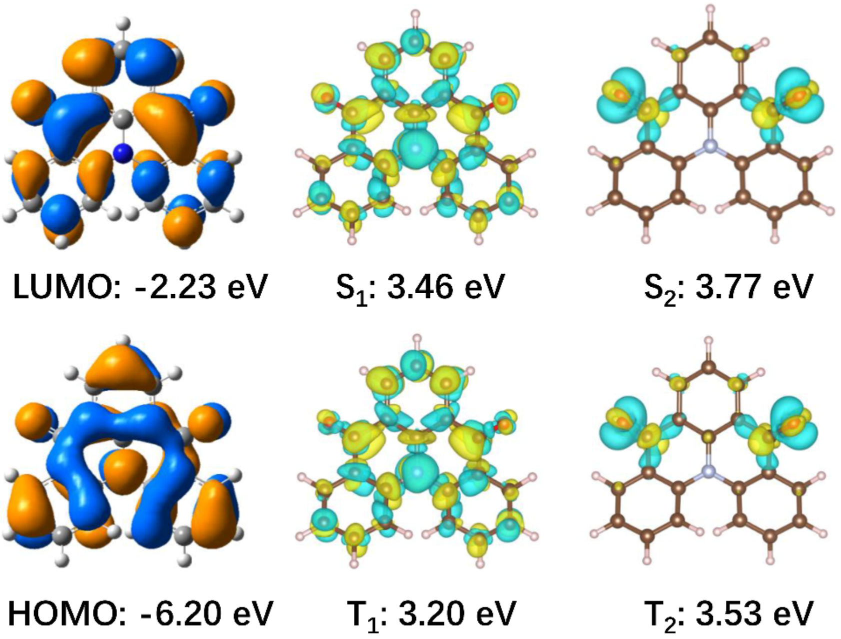

Figure **S4**. Theoretical calculations for **DiKTa**. HOMO and LUMO orbitals calculated in the gas phase at the PBE0/6-31G(d,p) level and difference density plots of S_1, S_2, T_1 and T_2 excited states calculated in the gas phase at the SCS-ADC(2)/cc-pVDZ level.

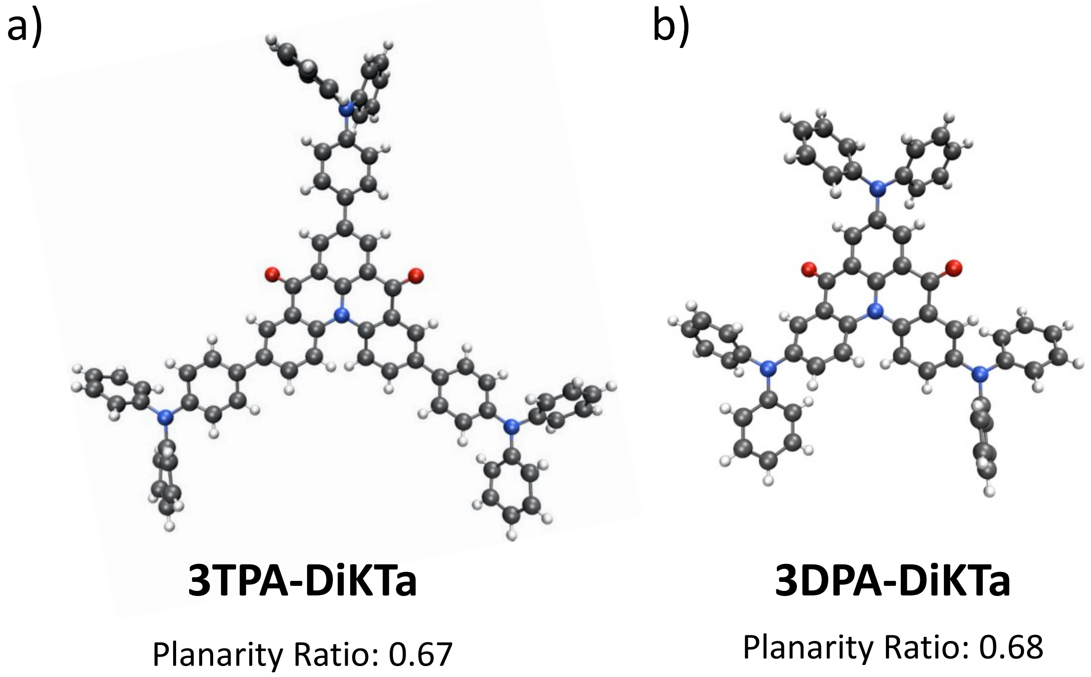

Figure **S5**. Planarity ratio, calculated at the PBE0/6-31G(d,p) level based on the crystal structure geometry of **3TPA-DiKTa** and **3DPA-DiKTa**.

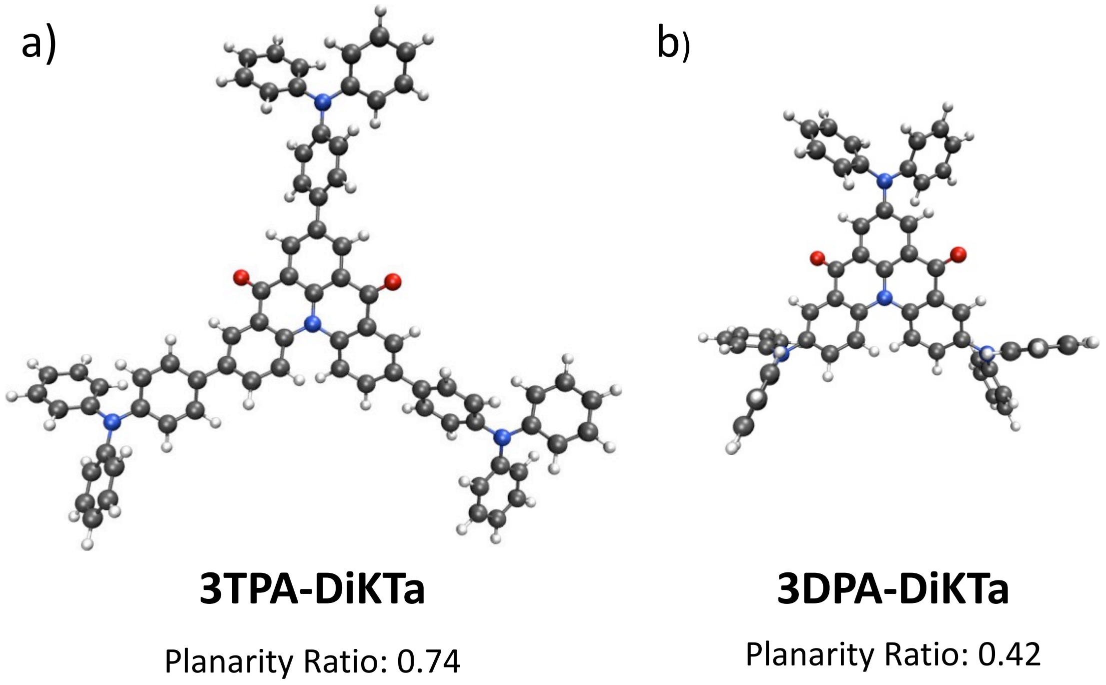

Figure **S6**. Planarity ratio, calculated at the PBE0/6-31G(d,p) level based on the DFT optimized structures of **3TPA-DiKTa** and **3DPA-DiKTa**.

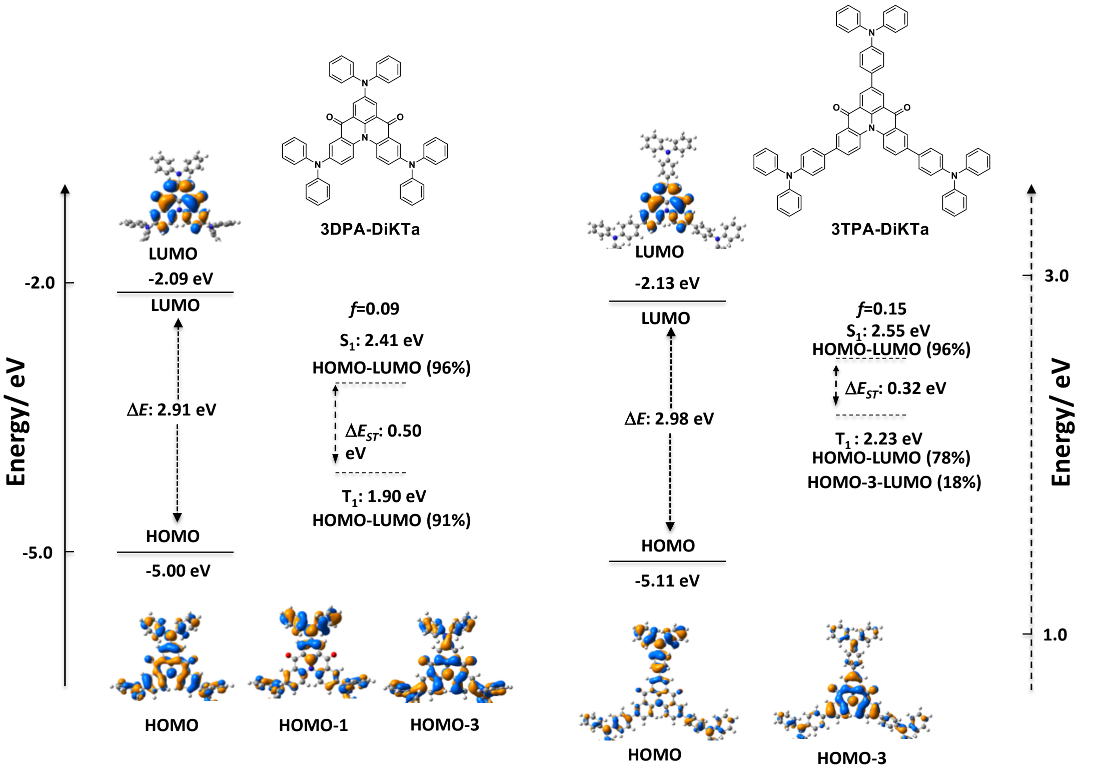

Figure **S7.** Distributions of the frontier molecular orbitals of **3TPA-DiKTa** and **3DPA-DiKTa**, calculated in the gas phase at the PBE0/6-31G(d,p) level, *f* is the oscillator strength.

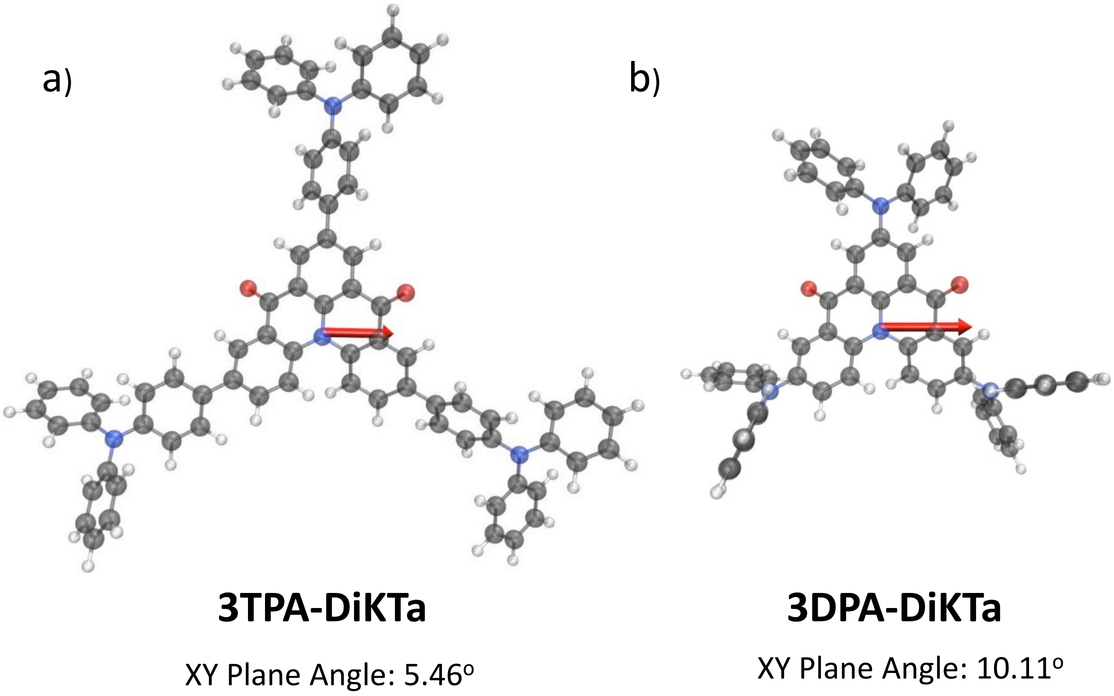

Figure **S8**. Molecular structures with transition dipole moments (denoted by red arrows) for **3TPA-DiKTa** and **3DPA-DiKTa**, calculated at the PBE0/6-31G(d) level.

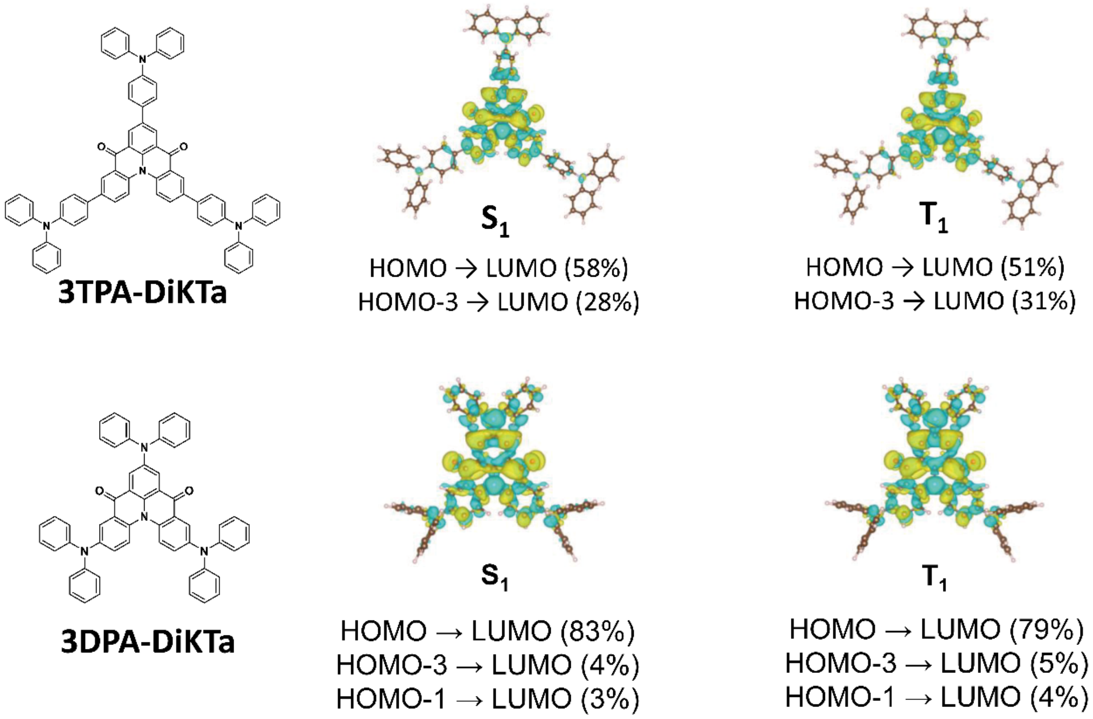

Figure **S9.** The electronic transition nature calculated using SCS-ADC(2)/cc-pVDZ in the gas phase for **3TPA-DiKTa** and **3DPA-DiKTa**.

Table **S2.** Calculated data in the gas phase at the PBE0/6-31G(d,p) level.

**Compound**	**FMOs / eV**	**S_1 /eV**	**T_1 / eV**	**ΔE_{ST} / eV**	***f***
**3TPA-DiKTa**	H: -5.10; H3: -6.02; L: -2.12	2.55	2.23	0.32	0.09
**3DPA-DiKTa**	H: -5.01; H3: -6.52; L: -2.09<br>H1: -5.38	2.41	1.90	0.50	0.15

Where H3=HOMO-3, H1=HOMO-1, H=HOMO，L=LUMO.

Table **S3.** Calculated data calculated in the gas phase at the ADC(2)/cc-pVDZ level.

**Compound**	**S_1/ eV (D_{CT} / Å)**	**S_2/ eV (D_{CT} / Å)**	**T_1/ eV (D_{CT} / Å)**	**T_2/ eV (D_{CT} / Å)**	**DE_{ST} / eV**	***f***
**3TPA-DiKTa**	3.16 (0.28)	3.65 (0.28)	2.93 (0.52)	-	0.23	0.29
**3DPA-DiKTa**	2.72 (0.20)	3.28 (0.11)	2.46 (0.11)	3.03 (0.39)	0.26	0.18

Where D_{CT} is the distance between the hole and electron density barycentre.

Synthesis

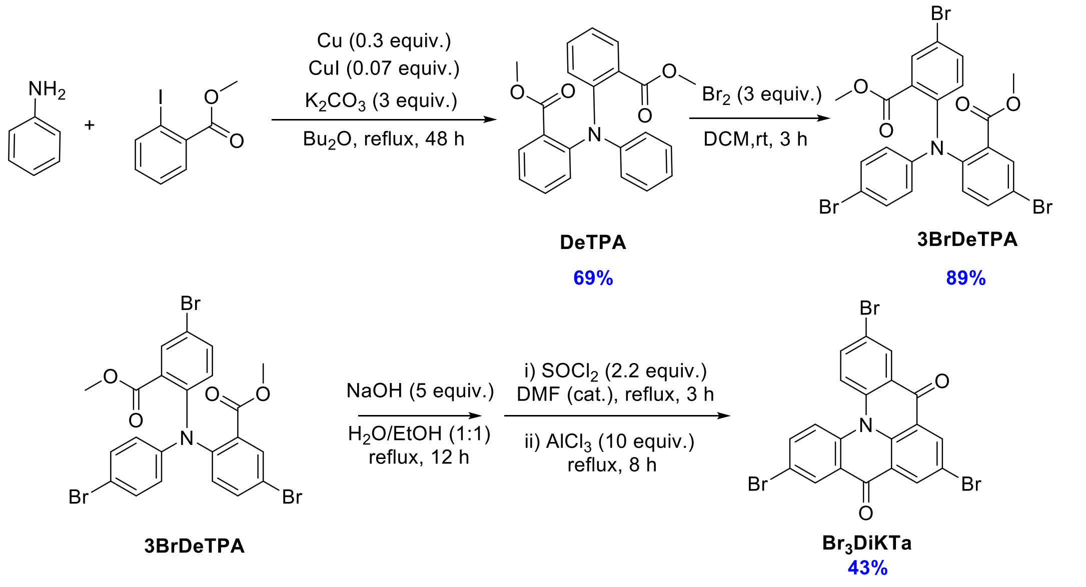

Scheme S1. Synthesis route of **Br_3DiKTa.**

Br_3DiKTa were synthesised in four steps as shown in Scheme **S1**, including Ullmann coupling, bromination, hydrolysis and Friedel-Craft acylation reaction. The detailed procedure was described in our previously reported protocol.[23]

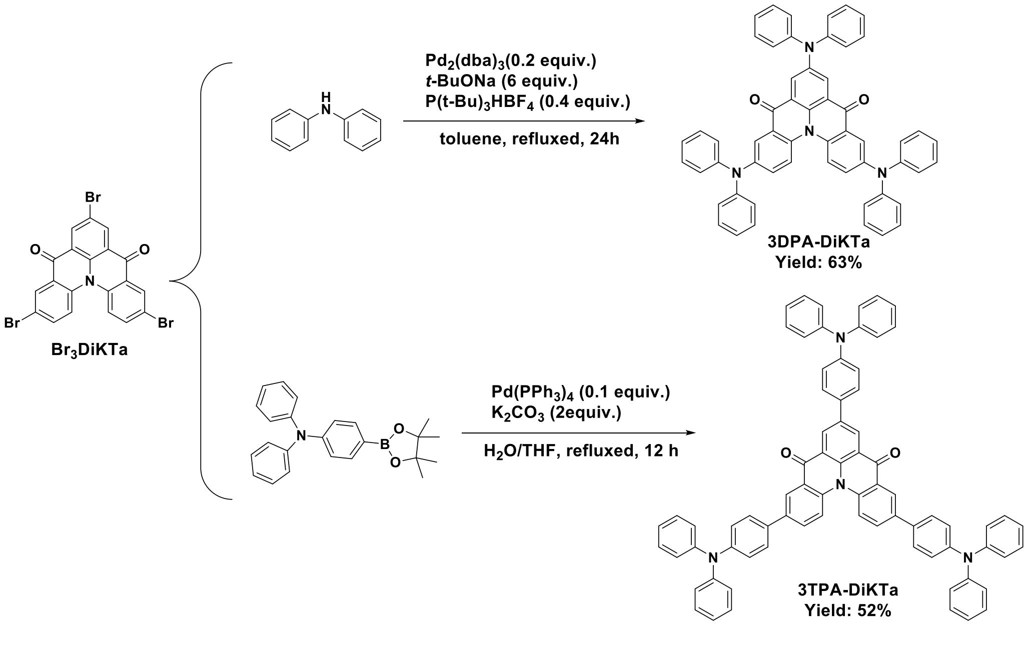

Scheme **S2**. Synthetic route for **3TPA-DiKTa** and **3DPA-DiKTa**.

3,7,11-tris(diphenylamino)quinolino[3,2,1-de]acridine-5,9-dione (3DPA-DiKTa)

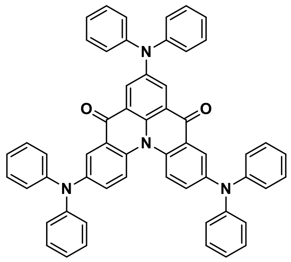

3DPA-DiKTa

Br_3DiKTa (600 mg, 1.12 mmol 1 equiv.), diphenylamine (856 mg, 5.06 mmol, 4.5 equiv.), $Pd_2(dba)_3$ (206 mg, 0.23 mmol, 0.2 equiv.), tri-*tert*-butylphosphonium tetrafluoroborate (91 mg, 0.45 mmol, 0.4 equiv.), sodium *tert*-butoxide (486 mg, 5.06 mmol, 4.5 equiv.) and 15 mL dry toluene were added to a 50 mL Schlenk tube, then placed under a nitrogen atmosphere. The solution was heated at reflux and stirred for 24 h. After cooling to room temperature, 50 mL DCM was added to the reaction mixture. The organic phase was collected and washed with brine (3×50 mL). The solvents were removed under reduced pressure. The crude product was purified by column chromatography on silica gel (EtOAc:hexane = 1:15). The corresponding fractions were combined and concentrated under reduced pressure to afford a deep red solid. **Yield:** 61% (550 mg). **R_f:** 0.35 (EtOAc:hexane =1:6). **Mp**: 287-289 ℃. **^{1}H-NMR** (500 MHz, $CDCl_3$) δ 8.44 (s, 2H), 8.04 (dd, *J* = 17.4, 6.0 Hz, 4H), 7.42 (dd, *J* = 9.2, 2.8 Hz, 2H), 7.31 (dt, *J* = 10.3, 5.1 Hz, 12H), 7.17 (d, *J* = 7.6 Hz, 8H), 7.14 – 7.09 (m, 8H), 7.08 (d, *J* = 7.4 Hz, 2H). **^{13}C-NMR** (126 MHz, $CDCl_3$) δ 177.70, 147.10, 146.04, 145.02, 144.17, 134.23, 134.15, 129.70, 129.05, 127.80, 127.06, 126.91, 124.73, 124.41, 124.31, 123.82, 123.76, 121.37, 119.29. **HRMS** (ESI-MS): **[$C_{56}H_{32}N_4O_2$ +Na]$^+$ Calculated:** 821.2887; **Found:** 821.2885. 97.87% pure by HPLC analysis, retention time 8.3 minutes in 95% Methanol and 5% Water. **Analysis calculated for** $C_{56}H_{38}N_4O_2$: C, 84.19%; H, 4.79%; N, 7.01 %; **Found.** C, 83.74%, H, 4.84% and 6.63%.

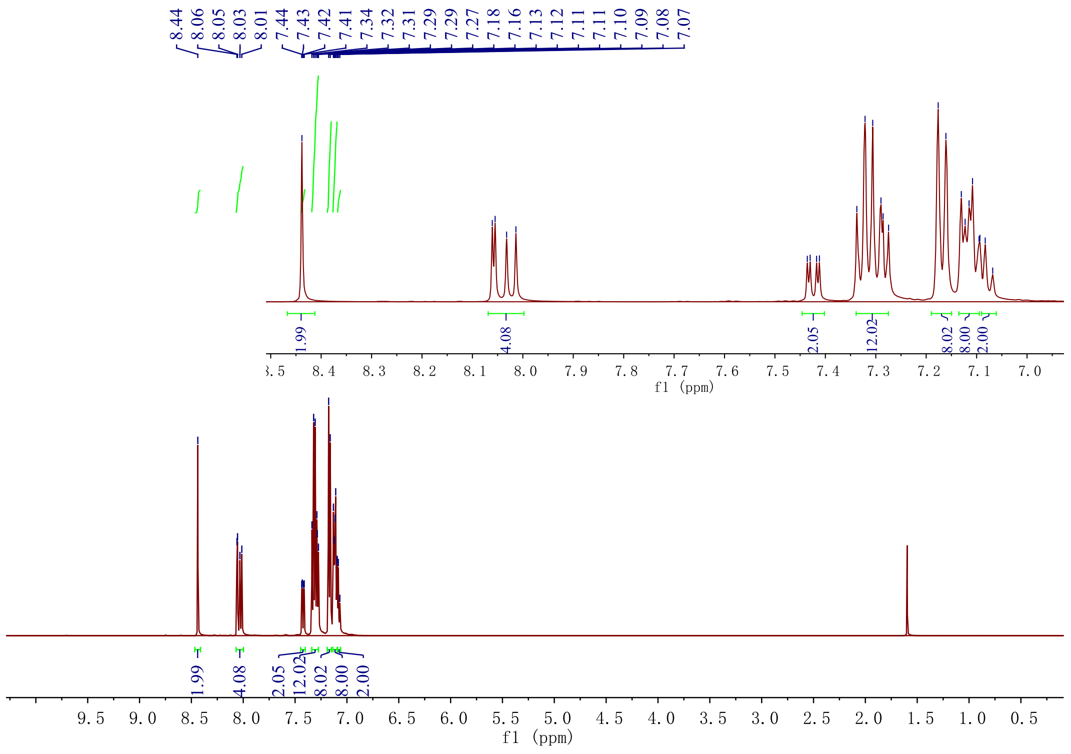

Figure **S10**. ^{1}H-NMR of **3DPA-DiKTa** in $CDCl_3$.

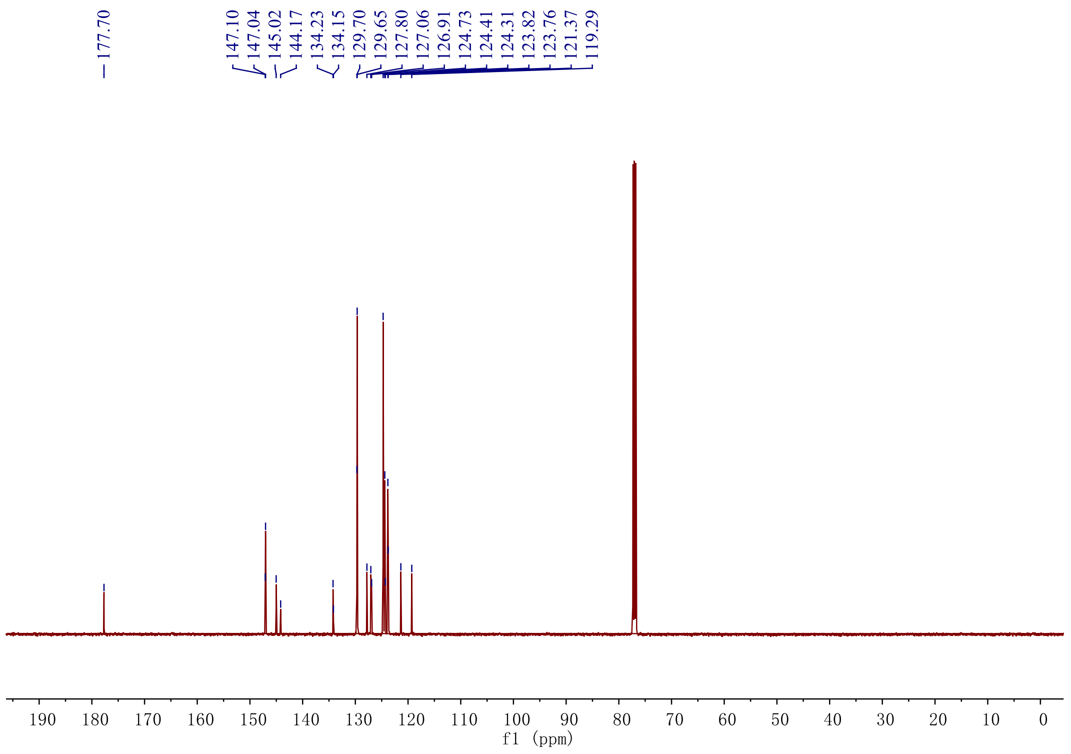

Figure **S11**. ^{13}C-NMR of **3DPA-DiKTa** in $CDCl_3$.

Display Report

Analysis Info

Analysis Name	D:\Data\Alans Data May 2021\STA_Jingxiang Wang 3TPA dIKTA.d	Acquisition Date	5/13/2021 10:24:19 AM
Method	010713alanstune_low.m	Operator	Bruker UK
Sample Name	Jingxiang Wang 3TPA dIKTA	Instrument	micrOTOF 8213750.10408
Comment	Jingxiang Wang 3TPA dIKTA		

Acquisition Parameter

Source Type	ESI	Ion Polarity	Positive			Set Nebulizer	1.0 Bar
Focus	Active					Set Dry Heater	210 °C
Scan Begin	40 m/z	Set Capillary	4500 V			Set Dry Gas	4.0 l/min
Scan End	3000 m/z	Set End Plate Offset	-500 V			Set Divert Valve	Waste

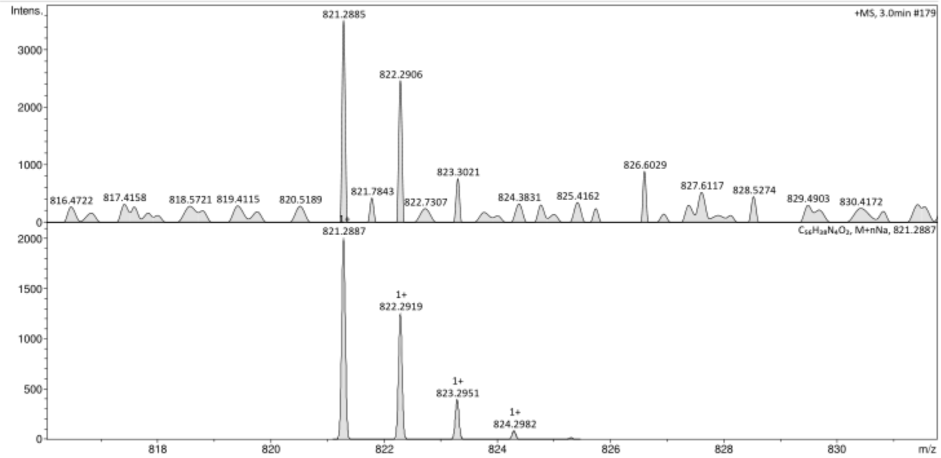

Bruker Compass DataAnalysis 4.1 printed: 5/13/2021 10:35:09 AM by: Bruker UK 1 of 1

Figure **S12**. HRMS of **3DPA-DiKTa**.

HPLC Trace Report03Mar2022

<Sample Information>

Sample Name	: 3dpa-2		
Sample ID	:		
Method Filename	: 95% Methanol 5 Water 20 mins.lcm		
Batch Filename	: 3dpa.lcb		
Vial #	: 1-9	Sample Type	: Unknown
Injection Volume	: 10 uL		
Date Acquired	: 03/03/2022 12:10:11	Acquired by	: System Administrator
Date Processed	: 03/03/2022 14:36:34	Processed by	: System Administrator

<Chromatogram>

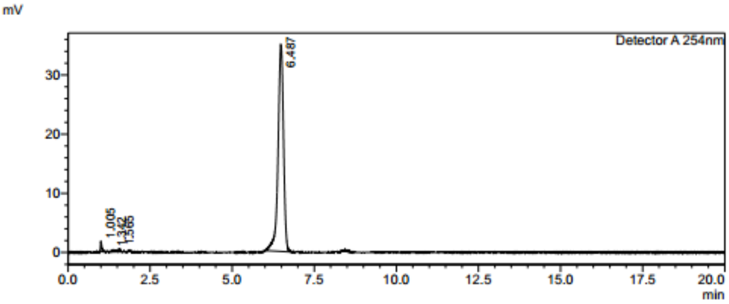

<Peak Table>

Detector A 254nm

Peak#	Ret. Time	Area	Height	Area%	Area/Height	Width at 5% Height
1	1.005	4826	1721	1.170	2.805	0.119
2	1.342	2245	269	0.544	8.357	--
3	1.565	1678	469	0.407	3.578	--
4	6.487	403725	34911	97.879	11.564	0.429
Total		412474	37369	100.000		

Figure **S13**. HPLC of **3DPA-DiKTa** with 95% Methanol and 5% water.

Elemental Analysis Service Request Form

Researcher name Sen Wu

Researcher email ws60@st-andrews.ac.uk

NOTE: Please submit ca. 10 mg of sample

**Sample reference number**	WS-I160
**Name of Compound**	3DPA-DiKTa
**Molecular formula**	C56H38N4O2
**Stability**	
**Hazards**	
**Other Remarks**	

Analysis type:

Single ☐ **Duplicate** ☑ **Triplicate** ☐

Analysis Result:

Element	Expected %	Found (1)	Found (2)	Found (3)
Carbon	84.19	83.49	83.74	
Hydrogen	4.79	4.79	4.84	
Nitrogen	7.01	6.60	6.63	
Oxygen				

Authorising Signature:

**Date completed**	15·12·21
**Signature**	J-P C—
**comments**	

Figure **S14**. Elemental analysis of **3DPA-DiKTa**.

3,7,11-tris(4-(diphenylamino)phenyl)quinolino[3,2,1-de]acridine-5,9-dione (3TPA-DiKTa)

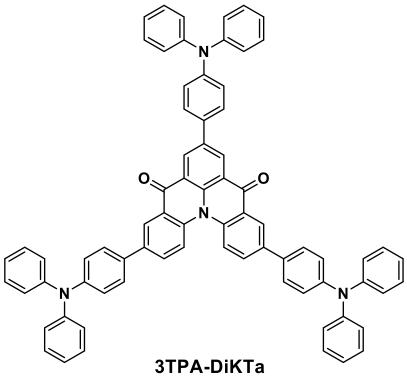

3TPA-DiKTa

Under a nitrogen atmosphere, to **Br3DiKTa** (400 mg, 0.75 mmol 1 equiv.) were added *N,N*-diphenyl-4-(4,4,5,5-tetramethyl-1,3,2-dioxaborolan-2-yl)aniline (973 mg, 2.62 mmol, 3.5 equiv.), THF (15 mL) and $NaOH_{aq}$ (2 M, 1.5 mL). Under a positive flow of

nitrogen, $Pd(PPh_3)_4$ (43 mg, 0.05 mmol, 0.05 equiv.) was added and the solution was then heated at reflux for 12 h. The reaction was cooled to room temperature and diluted with DCM (150 mL). The organic layer was washed with water (3 × 50 mL) and then dried with anhydrous sodium sulfate. The solvents were removed under reduced pressure. The crude product was purified by column chromatography on silica gel (EtOAc:hexane = 1:15). The corresponding fractions were combined and concentrated under reduced pressure to afford bright yellow solid. **Yield:** 52% (400 mg) **R_f:** 0.30 (EtOAc:hexane = 1:5) **Mp** > 400℃. **^{1}H-NMR** (500 MHz, $CDCl_3$) δ 9.00 (s, 2H), 8.73 (d, J = 2.2 Hz, 2H), 8.25 (d, J = 8.9 Hz, 2H), 7.95 (dd, J = 8.8, 2.2 Hz, 2H), 7.72 (d, J = 8.6 Hz, 2H), 7.65 (d, J = 8.6 Hz, 4H), 7.33 (t, J = 7.8 Hz, 12H), 7.24 – 7.17 (m, 18H), 7.10 (t, J = 7.3 Hz, 6H). **^{13}C-NMR** (126 MHz, $CDCl_3$) δ 178.69, 148.07, 147.98, 147.48, 138.25, 137.79, 137.51, 136.17, 132.31, 131.86, 130.76, 130.31, 129.41, 127.85, 127.65, 126.58, 124.77, 123.86, 123.53, 123.33, 120.92. **HRMS** (ESI-MS): **$[C_{74}H_{50}N_4O_2 +H]^+$ Calculated:** 1027.4007; **Found:** 1027.3996. **Analysis calculated for** $C_{74}H_{50}N_4O_2$: C, 86.52%; H, 4.91%; N, 5.45%; **Found.** C, 86.94, H, 4.90% and 5.53%.

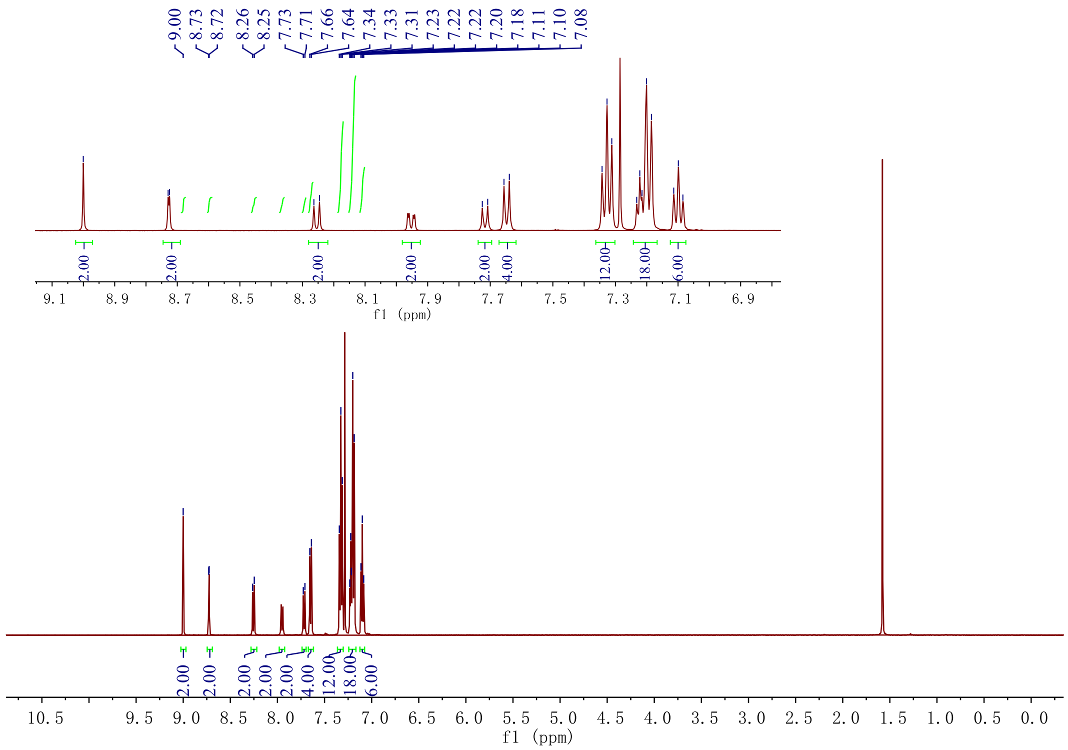

Figure **S15**. ^{1}H-NMR of **3TPA-DiKTa** in $CDCl_3$.

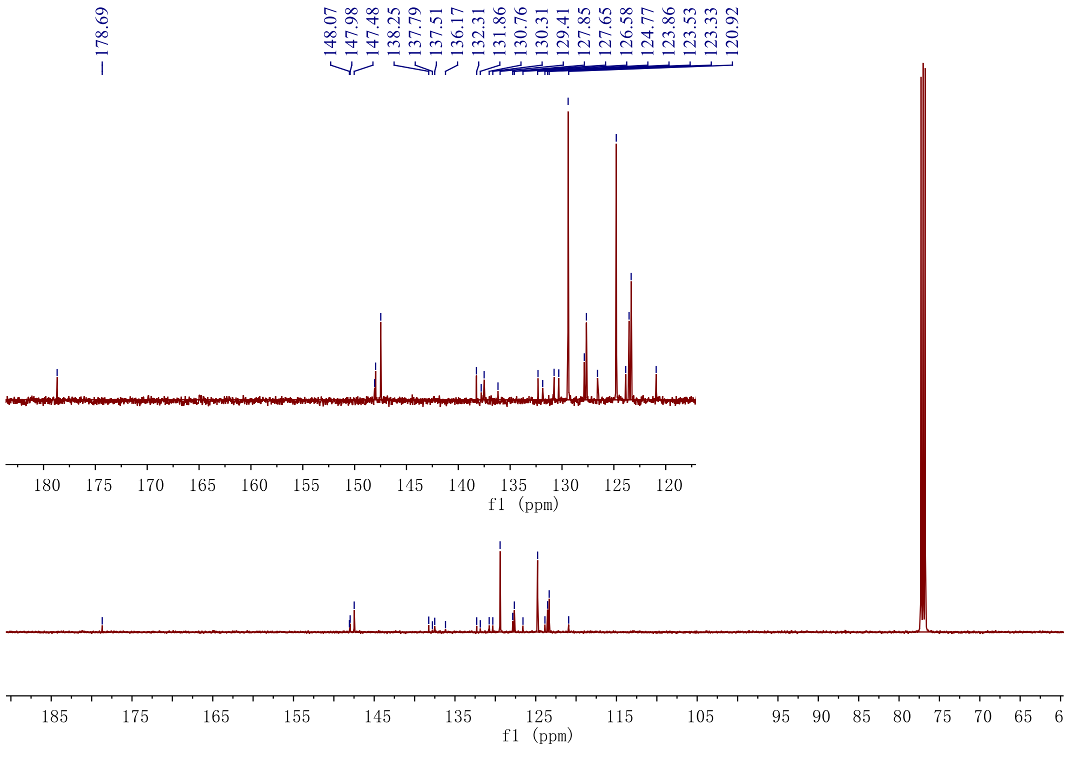

Figure **S16.** ^{13}C-NMR of **3TPA-DiKTa** in $CDCl_3$.

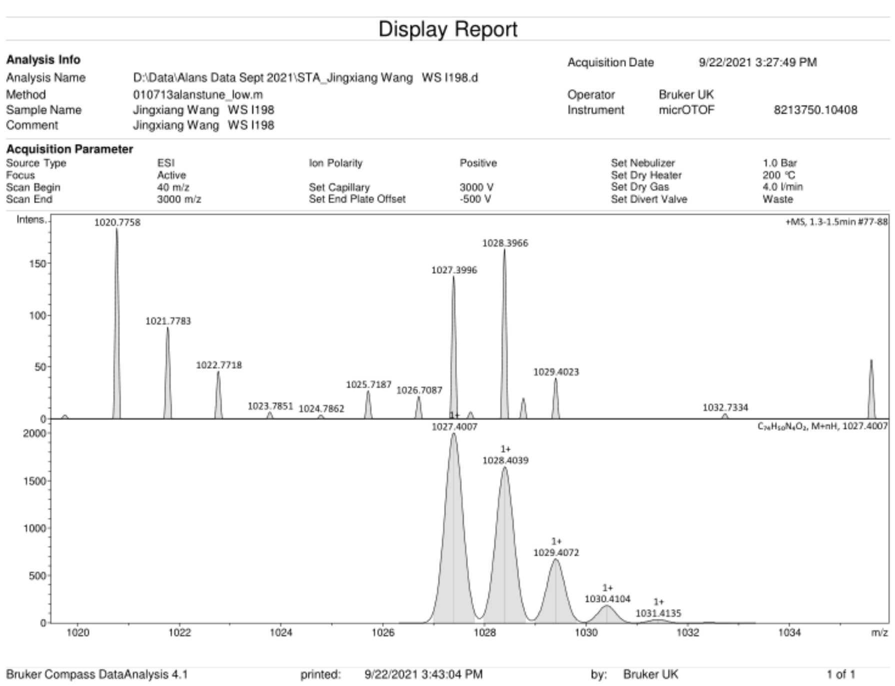

Figure **S17.** HRMS of **3TPA-DiKTa**.

**Molecular formula**	C74H50N4O2
**Stability**	Stable in air and in most solvents
**Hazards**	
**Other Remarks**	

Analysis type:

Single ☐ **Duplicate** ■ **Triplicate** ☐

Analysis Result:

Element	Expected %	Found (1)	Found (2)	Found (3)
Carbon	86.52	86.94	85.04	
Hydrogen	4.91	4.90	4.89	
Nitrogen	5.45	5.53	5.37	
Oxygen				

Authorising Signature:

**Date completed**	21.03.22
**Signature**	J-PC
**comments**	

Figure **S18.** Elemental Analysis of **3TPA-DiKTa**.

Optoelectronic characterization

Table **S4.** Electrochemical data

	E_{ox} /V[a]	E_{red} /V[a]	HOMO / eV[b]	LUMO /eV[b]
**DiKTa**	1.78	-1.34	-6.12	-3.00
**3TPA-DiKTa**	0.93	-1.36	-5.27	-2.98
**3DPA-DiKTa**	0.79	-1.33	-5.13	-3.01

E_{ox} and E_{red} are the peak of anodic and cathodic potentials from DPV relative to Fc/Fc$^+$. In degassed DCM with 0.1 M [nBu$_4$N]PF$_6$ as the supporting electrolyte and Fc/Fc$^+$ as the internal reference (0.46 V vs. SCE).[1, 24] $^b E_{HOMO/LUMO} = -(E_{ox} / E_{red} + 4.8)$ eV.

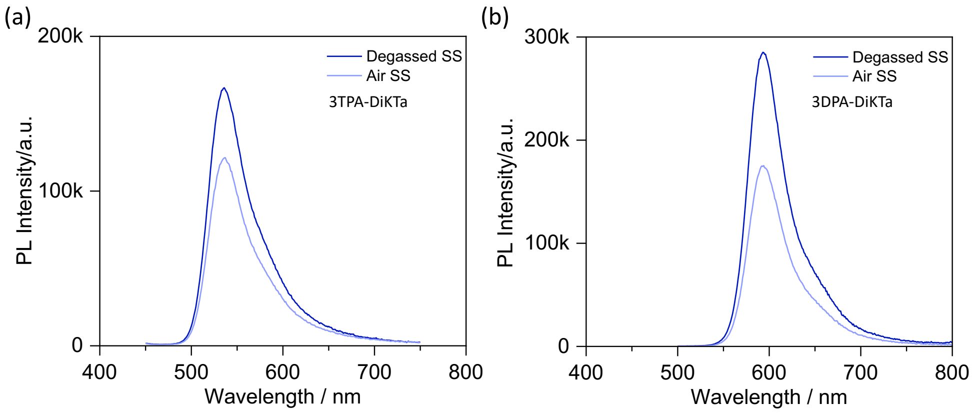

Figure **S19**. Comparison of the steady-state PL in both aerated and degassed toluene of (a) **3TPA-DiKTa** and (b) **3DPA-DiKTa** (λ_{exc} = 340 nm).

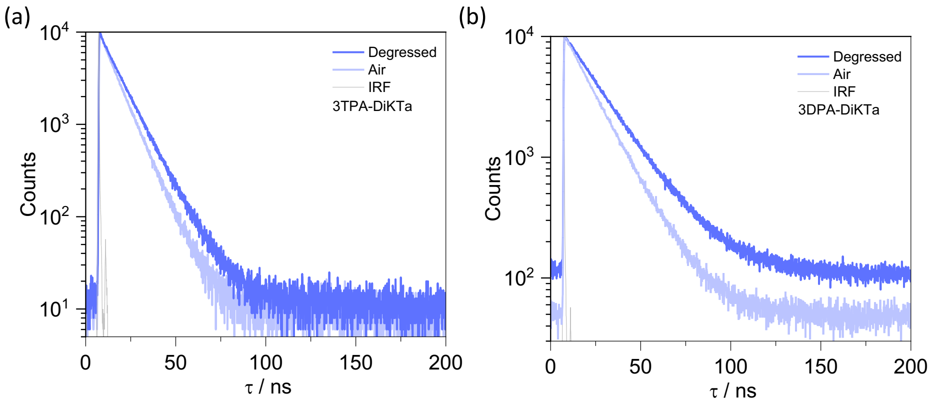

Figure **S20**. Comparison of PL decay in both aerated and degassed toluene of (a) **3TPA-DiKTa** and (b) **3DPA-DiKTa** (λ_{exc} = 375 nm).

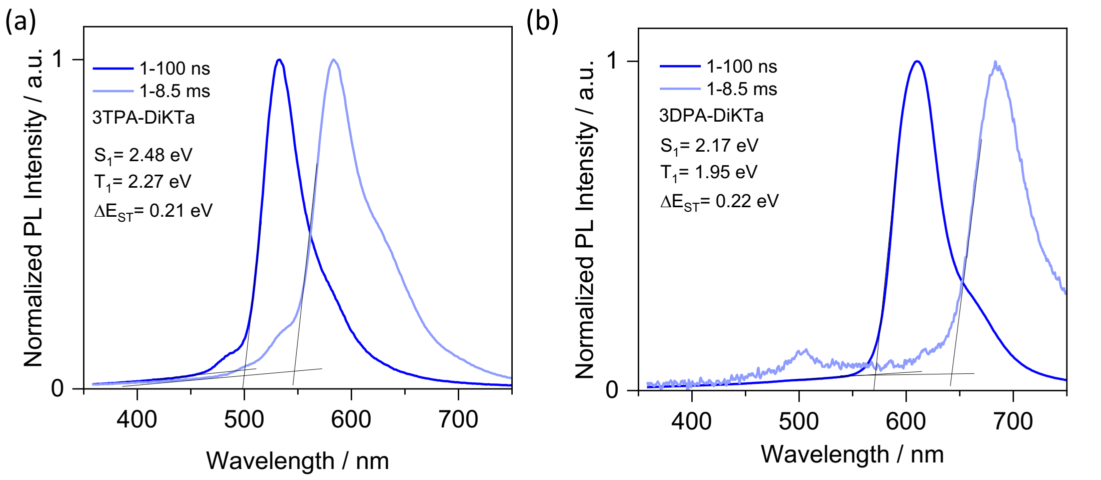

Figure **S21**. Prompt PL (1-100 ns) and phosphorescence spectra (1-8.5 ms) obtained in 2-MeTHF glass at 77 K for (a) **3TPA-DiKTa** and (b) **3DPA-DiKTa** (λ_{exc} = 343 nm).

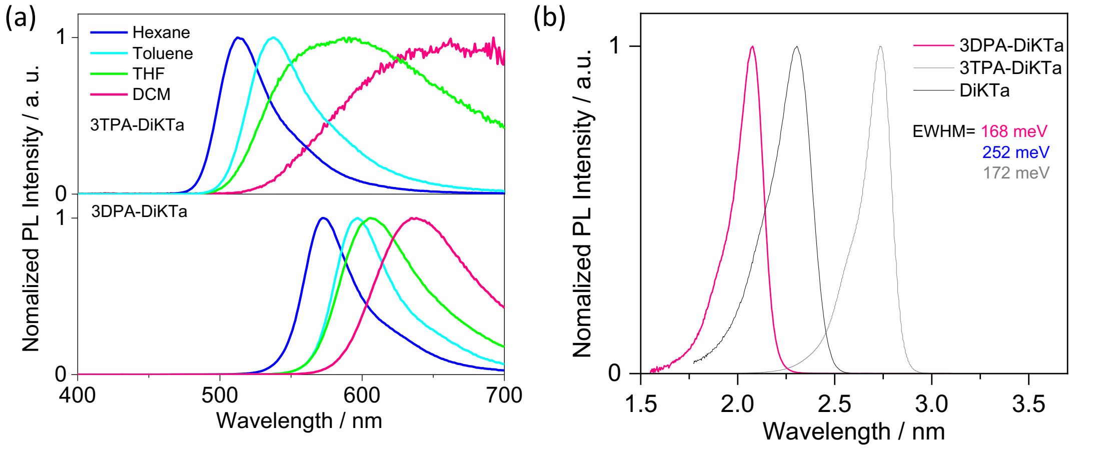

Figure **S22.** (a) Solvatochromatic PL study for **3TPA-DiKTa** and **3DPA-DiKTa**. (λ_{exc} = 340 nm), (b) The PL spectra of **DiKTa**, **3TPA-DiKTa** and **3DPA-DiKTa** after Jacobian transformation.[25]

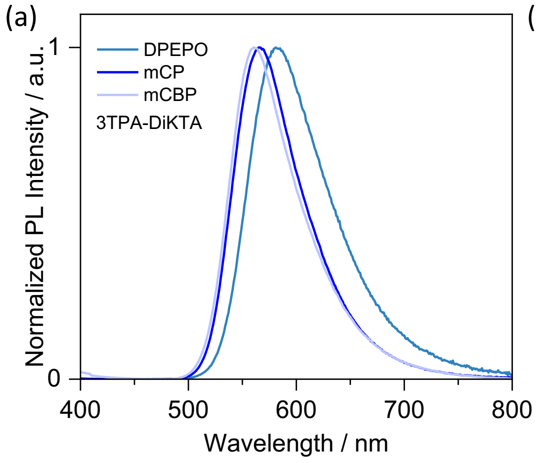

Figure **S23**. PL of doped film in different host matrices (2 wt%) for (a) **3TPA-DiKTa** and (b) **3DPA-DiKTa**, (λ_{exc} = 340 nm).

Table **S5.** Photoluminescence quantum yield screening in different host matrices.

	**PLQY**[b]		
**Host**[a]	**mCP**	**mCBP**	**DPEPO**
**3TPA-DiKTa**	87% (68%)	75% (67%)	64% (54%)
**3DPA-DiKTa**	60% (52%)	-	46% (37%)

[a] Thin films were prepared by spin-coating with 2 wt% in each host; [b] Φ_{PL} values were determined using an integrating sphere (λ_{exc} = 305 nm or 340 nm); degassing was done by N_2 purge (value given inside parentheses in the presence of O_2). Φ_{PL} values are within an error limit of ± 2%.

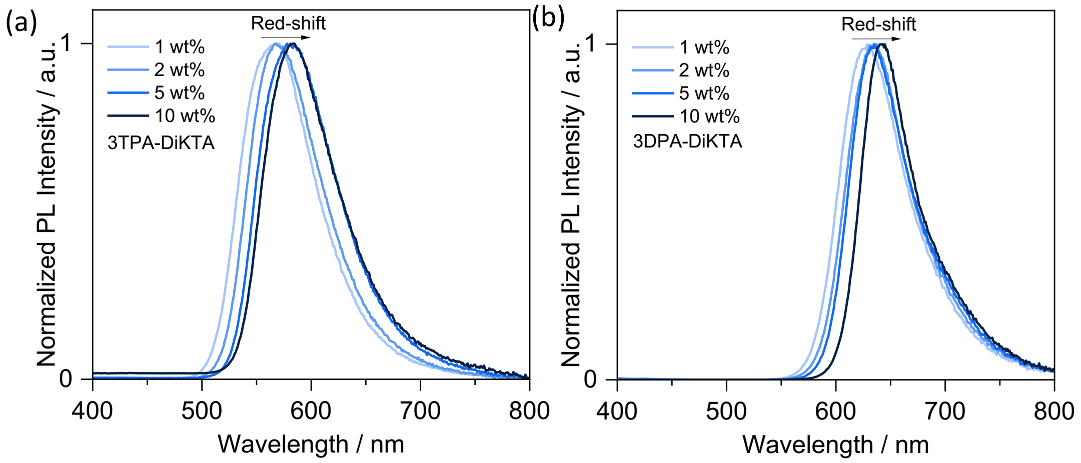

Figure **S24**. Concentration-dependent PL of (a) **3TPA-DiKTa** and (b) **3DPA-DiKTa** in mCP, λ_{exc} = 340 nm.

Table **S6.** Photoluminescence quantum yield screening at different doping concentrations in spin-coated mCP thin films.[a]

Φ_{PL}[a]	1 wt%	2 wt%	5 wt%	10 wt%
**3TPA-DiKTa**	81% (69%)	87% (68%)	76% (68%)	71% (66%)
**3DPA-DiKTa**	65% (55%)	60% (52%)	51% (44%)	49% (42%)

[a] Φ_{PL} values were determined using an integrating sphere (λ_{exc} = 305 nm or 340 nm); degassing was done by N_2 purge (value given inside parentheses in the presence of O_2). Φ_{PL} values are within an error limit of ± 2%.

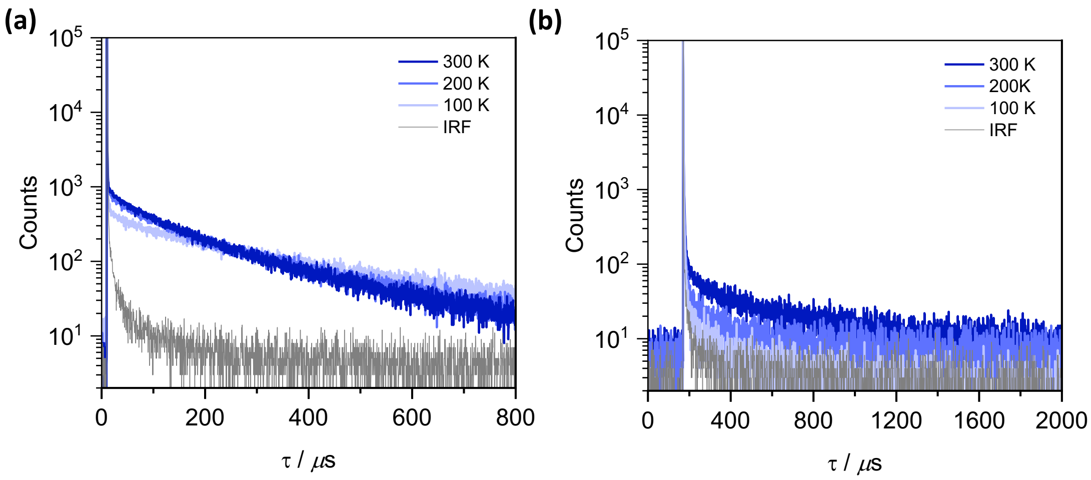

Figure **S25**. Temperature-dependent time-resolved PL decay of (a) **3TPA-DiKTa** and (b) **3DPA-DiKTa** in 2 wt% mCP, λ_{exc} = 379 nm.

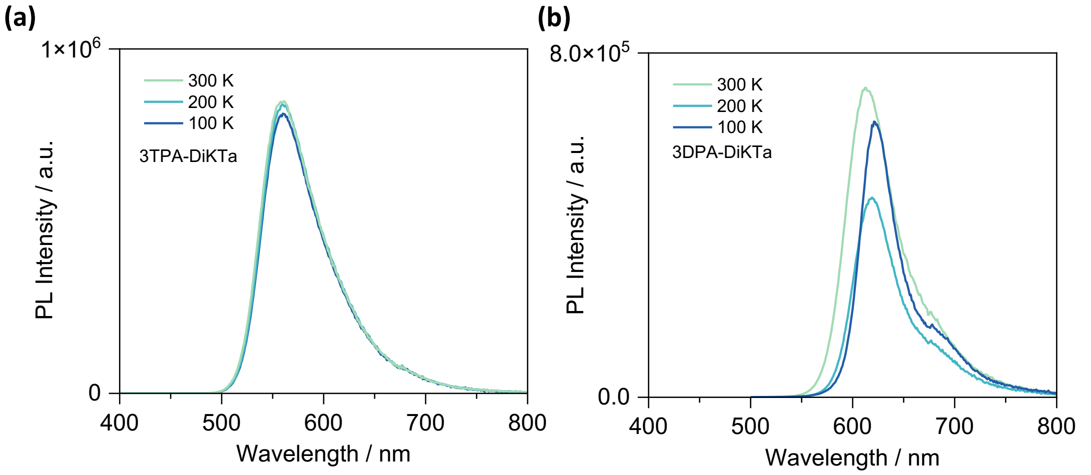

Figure **S26**. Temperature-dependent steady-state PL spectra in 2 wt% mCP of (a)

3TPA-DiKTa and (b) **3DPA-DiKTa**. λ_{exc} = 340 nm.

For a TADF system, the main exciton loss channels are either singlet or triplet nonradiative transition processes. Owing to high performance, the singlet nonradiative transition process (k_{nr}^S) can be ignored, therefore the exciton loss can be attributed to the triplet nonradiative transition process (k_{nr}^T). The kinetic parameters were calculated according to the following equations and summarized in Table S7.[26]

$$\emptyset_{PL} = \emptyset_p + \emptyset_d \quad (1)$$

$$k_P = \frac{1}{\tau_p} \quad (2)$$

$$k_d = \frac{1}{\tau_d} \quad (3)$$

$$k_r^S = k_p \emptyset_p \quad (4)$$

$$k_{ISC} = k_p(1 - \emptyset_p) \quad (5)$$

$$k_{RISC} = \frac{k_p k_d}{k_{ISC}} \frac{\emptyset_d}{\emptyset_p} \quad (6)$$

$$k_{nr}^T = k_d - \emptyset_p k_{RISC} \quad (7)$$

Where the Φ_p and Φ_d are the prompt fluorescent and delayed fluorescent quantum efficiency; k_p is the rate constant of prompt fluorescence; k_d is the rate constant of delayed fluorescence; kr^S is the radiative decay rate constant of S_1; k_{nr}^T is the non-radiative decay rate constant of T_1; k_{ISC} is the intersystem crossing rate constant; k_{RISC} is the reverse intersystem crossing rate constant.

Table **S7.** Summary of kinetics parameters of 2 wt% films of **3TPA-DiKTa** and 3**DPA-DiKTa** in mCP.

**Compounds**	**Φ_P /%**	**Φ_d /%**	**k_p [10^7 s^{-1}]**	**k_d [10^3 s^{-1}]**	**k_r^S [10^7 s^{-1}]**	**k_{nr}^T [10^3 s^{-1}]**	**k_{ISC} [10^7 s^{-1}]**	**k_{RISC} [10^4 s^{-1}]**
**3DPA-DiKTa**	49	11	6.25	3.10	3.06	2.43	3.19	0.14
**3TPA-DiKTa**	28	65	7.14	7.63	1.98	0.74	5.17	2.49

OLED results:

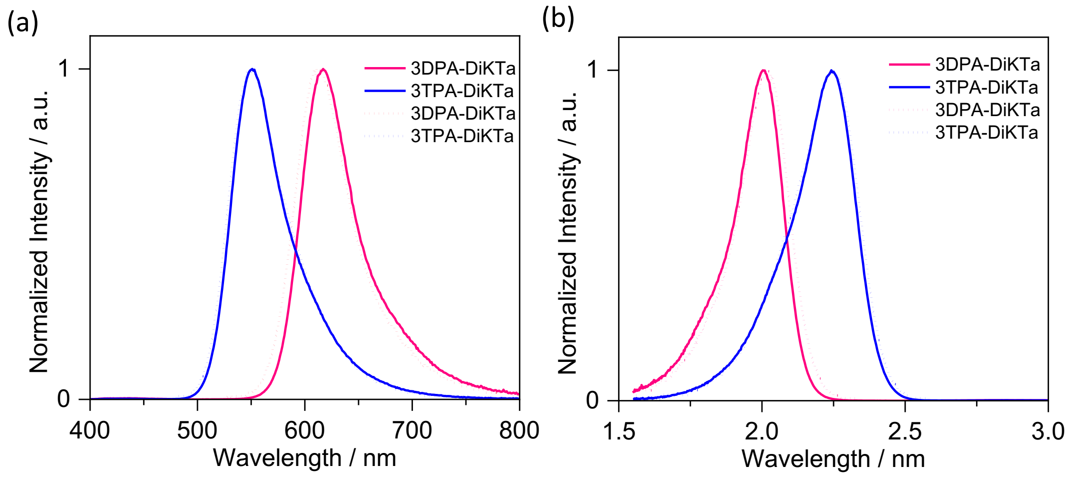

Figure **S27**. The comparation of PL (short dot) and EL (solid) spectra in 2 wt% doped film: (a) Normal spectra and (b) Jacobian transformation spectra.

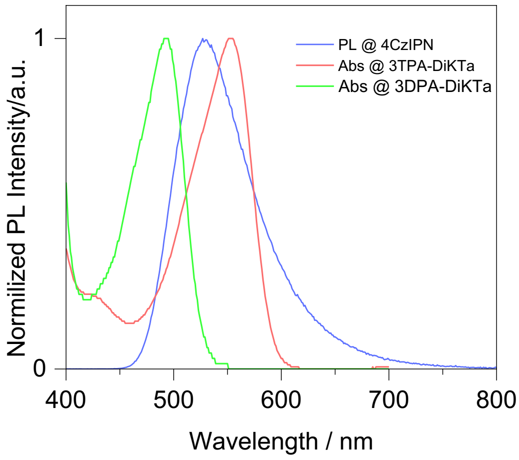

Figure **S28**. UV/vis absorption spectrum of **3DPA-DiKTa** and **3TPA-DiKTa** in toluene at a concentration of 10^{-5} M and PL spectrum of 10 wt% 4CzIPN : mCP doped film.

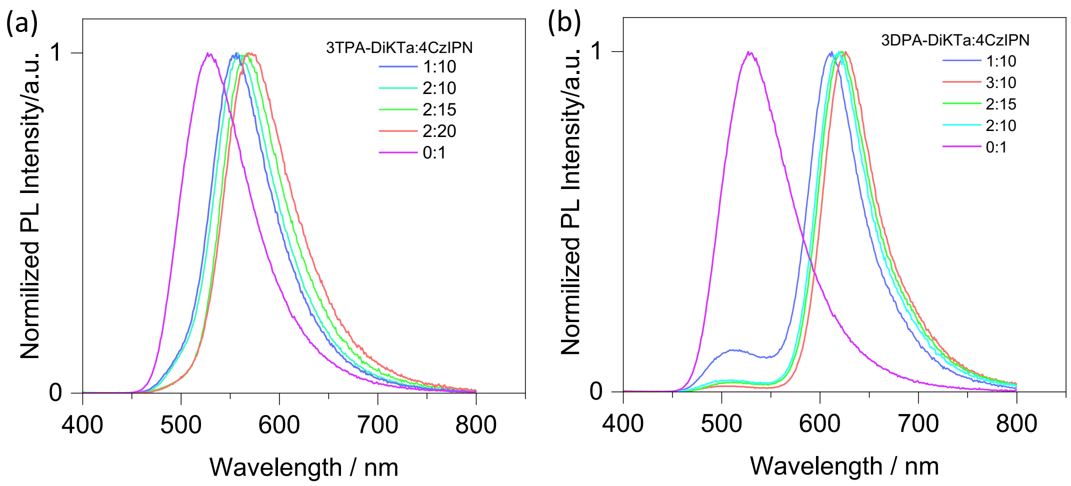

Figure **S29**. PL spectra of different doped concentration films of **3TPA-DiKTa** and **3DPA-DiKTa** with 4CzIPN in mCP host.

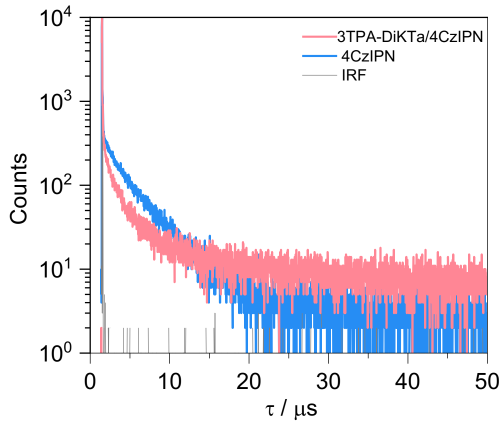

Figure **S30**. Time-resolved PL decay of 2 wt% **3TPA-DiKTa**:10 wt% 4CzIPN: mCP doped film.

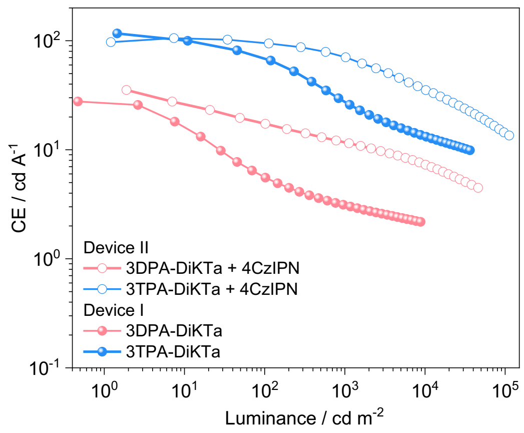

Figure **S31.** Current efficiency versus luminance curves for devices of **3DPA-DiKTa** and **3TPA-DiKTa** emitters in mCP host fabricated by thermal evaporation. Device **I** stack: ITO/HATCN (5 nm)/TAPC (40 nm)/TCTA (10 nm)/mCP (10 nm)/emissive layer (2 wt% emitter in mCP, 20 nm)/TmPyPB (50 nm)/LiF (0.6 nm)/Al (100 nm). Device **II** stack: ITO/HATCN (5 nm)/TAPC (40 nm)/TCTA (10 nm)/mCP (10 nm)/emissive layer (10wt% 4CzIPN and 2 wt% emitter in mCP, 20 nm)/TmPyPB (50 nm)/LiF (0.6 nm)/Al (100 nm)

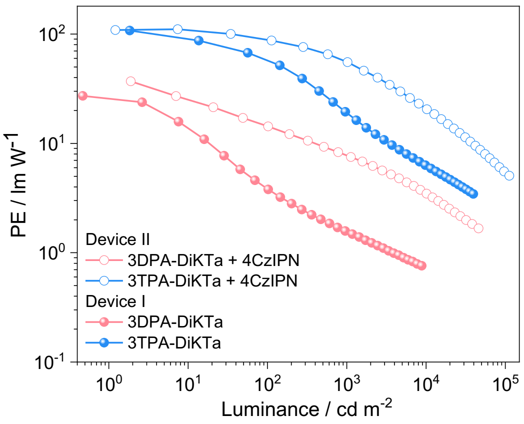

Figure **S32**. Power efficiency versus luminance curves for devices of **3DPA-DiKTa** and **3TPA-DiKTa** emitters in mCP host fabricated by thermal evaporation. Device **I** stack: ITO/HATCN (5 nm)/TAPC (40 nm)/TCTA (10 nm)/mCP (10 nm)/emissive layer (2 wt% emitter in mCP, 20 nm)/TmPyPB (50 nm)/LiF (0.6 nm)/Al (100 nm). Device **II** stack: ITO/HATCN (5 nm)/TAPC (40 nm)/TCTA (10 nm)/mCP (10 nm)/emissive layer (10wt% 4CzIPN and 2 wt% emitter in mCP, 20 nm)/TmPyPB (50 nm)/LiF (0.6 nm)/Al (100 nm)

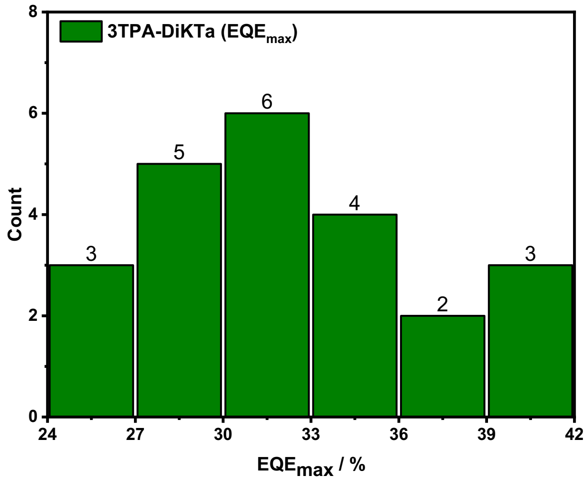

Figure **S33**. EQE histogram of 23 **3TPA-DiKTa**. Device stack; ITO/HATCN (5 nm)/TAPC (40 nm)/TCTA (10 nm)/mCP (10 nm)/emissive layer (20 nm)/TmPyPB (50 nm)/LiF (0.6 nm)/Al (100 nm).

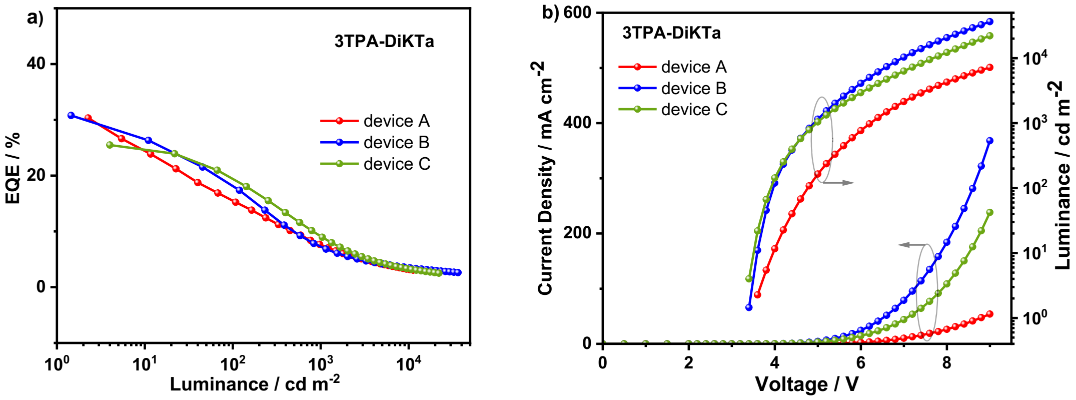

Figure **S34**. (a) External quantum efficiency versus luminance curves for the **3TPA-DiKTa** devices (A-C); (b) Current density and luminance versus voltage characteristics for the **3TPA-DiKTa** devices (A-C). **Device A**: ITO/HATCN (5 nm)/TAPC (40 nm)/ mCP (10 nm)/emissive layer (2 wt% **3TPA-DiKTa** in mCP) (20 nm)/PPT (10 nm)/TmPyPB (50 nm)/LiF (0.6 nm)/Al (100 nm). **Device B**: ITO/HATCN (5 nm)/TAPC (40 nm)/TCTA (10 nm)/mCP (10 nm)/emissive layer (2 wt% **3TPA-DiKTa** in mCP) (20 nm)/TmPyPB (50 nm)/LiF (0.6 nm)/Al (100 nm). **Device C**: ITO/HATCN (5 nm)/TAPC (40 nm)/TCTA (10 nm)/mCP (10 nm)/emissive layer (2 wt% **3TPA-DiKTa** in mCP) (20 nm)/PPT (10 nm)/TmPyPB (50 nm)/LiF (0.6 nm)/Al (100 nm).

We also fabricated devices A and C from **3TPA-DiKTa** by using TCTA (as a HTL)

and 2,8-bis(diphenyl-phosphoryl)-dibenzo[b,d]thiophene (PPT) (as a HBL). The OLEDs performances are shown in Figure **S34** and summarized in Table **S8**. Devices A and C reached lower luminance and low current density with poorer device performance than the optimized device structure (device B) (Figure **S34** and Table **S8**).

Table **S8.** Electroluminescence data of **3TPA-DiKTa** in the various device stacks.

**Devices**[a]	**V_{on}**[b]**/ V**	**EQE**[c] **/ %**	**L_{max}**[d]**/ cd m^{-2}**	**Current density**[d] **/ mA cm^{-2}**
**Device A**	3.6	30.3/16.0/7.6/3.1	11267	54
**Device B**	3.4	30.8/18.1/7.3/3.5	36421	368
**Device C**	3.4	25.5/19.3/9.1/3.3	22083	238

[a] **Device A**: ITO/HATCN (5 nm)/TAPC (40 nm)/mCP (10 nm)/emissive layer (2 wt% **3TPA-DiKTa** in mCP) (20 nm)/PPT (10 nm)/TmPyPB (50 nm)/LiF (0.6 nm)/Al (100 nm); **Device B**: ITO/HATCN(5 nm)/TAPC (40 nm)/TCTA (10 nm)/mCP (10 nm)/emissive layer (2 wt% **3TPA-DiKTa** in mCP) (20 nm)/TmPyPB (50 nm)/LiF (0.6 nm)/Al (100 nm); **Device C**: ITO/HATCN (5 nm)/TAPC (40 nm)/TCTA (10 nm)/mCP (10 nm)/emissive layer (2 wt% **3TPA-DiKTa** in mCP) (20 nm)/PPT (10 nm)/TmPyPB (50 nm)/LiF (0.6 nm)/Al (100 nm); [b] The turn-on voltage at EQE_{max}. [c] The order of measured values: the $EQE_{max}/EQE_{100}/EQE_{1000}/EQE_{10,000}$. [d] $Luminance_{max}$ and current density at 9 V.

Table **S9.** Performance of OLEDs using DiKTa derivatives

**Emitters**[a]	**λ_{EL} / nm**	**FWHM / nm**	**EQE_{max} / %**	**EQE_{100} / %**	**Roll-off / % at EQE_{100}**	**L_{max} / cd m^{-2}**	**Reference**
**3DPA-DiKTa**	613	60	16.7	3.4	79.6	8829	**This work**
**3TPA-DiKTa**	551	62	30.8	18.1	41.2	36 421	**This work**
**3DPA-DiKTa (HF)**	615	61	17.9	8.7	51.4	46 003	**This work**
**3TPA-DiKTa (HF)**	556	70	30.0	27.4	8.6	112 190	**This work**
**DiKTa**	465	39	14.7	8.3	43.5	10 385	[27]
**Mes3DiKTa**	480	36	21.1	14.5	31.3	12 949	
**QAD (akaDiKTa)**	468	39	19.4	9.4	51.5	1100	[28]
**3Ph-QAD**	480	44	19.1	10.4	45.5	4975	[29]
**7Ph-QAD**	472	34	18.7	2.1	88.7	2944	
**Cz-DiKTa**	511	62	24.9	20.4	18.1	13 260	[8]
**Cz-Ph-DiKTa**	492	61	23.0	19.3	16.1	8529	
**3Cz-DiKTa**	547	54	24.4	17.3	29.1	10 796	
**QAD-Cz**	494	57	20.3	5.4	73.4	-	[30]

**QAD-2Cz**	530	56	27.3	23.9	12.4	-	
**QAD-mTDPA**	589	67	26.3	12.9	51.0	-	
**DDiKTa**	500	59	19.0	7.9	58.4	501	[31]
**QA-PF**	474	27	16.8	5.6	66.6	1740	
**QA-PCN**	473	30	16.9	9.4	44.4	2760	[32]
**QA-PMO**	484	27	15.0	3.5	76.6	3040	
**QA-PCZ**	482	29	17.5	7.6	56.5	3600	
**QAOCz1**	516	44	16.9	-	-	11320	
**QAOCz2**	504	43	19.4	-	-	7679	[33]
**QAOCz3**	500	40	21.1	-	-	6217	
**BOQAO**	484	32	21.8	-	-		[34]

[a] Emitter structures are shown in Figure **S32**.

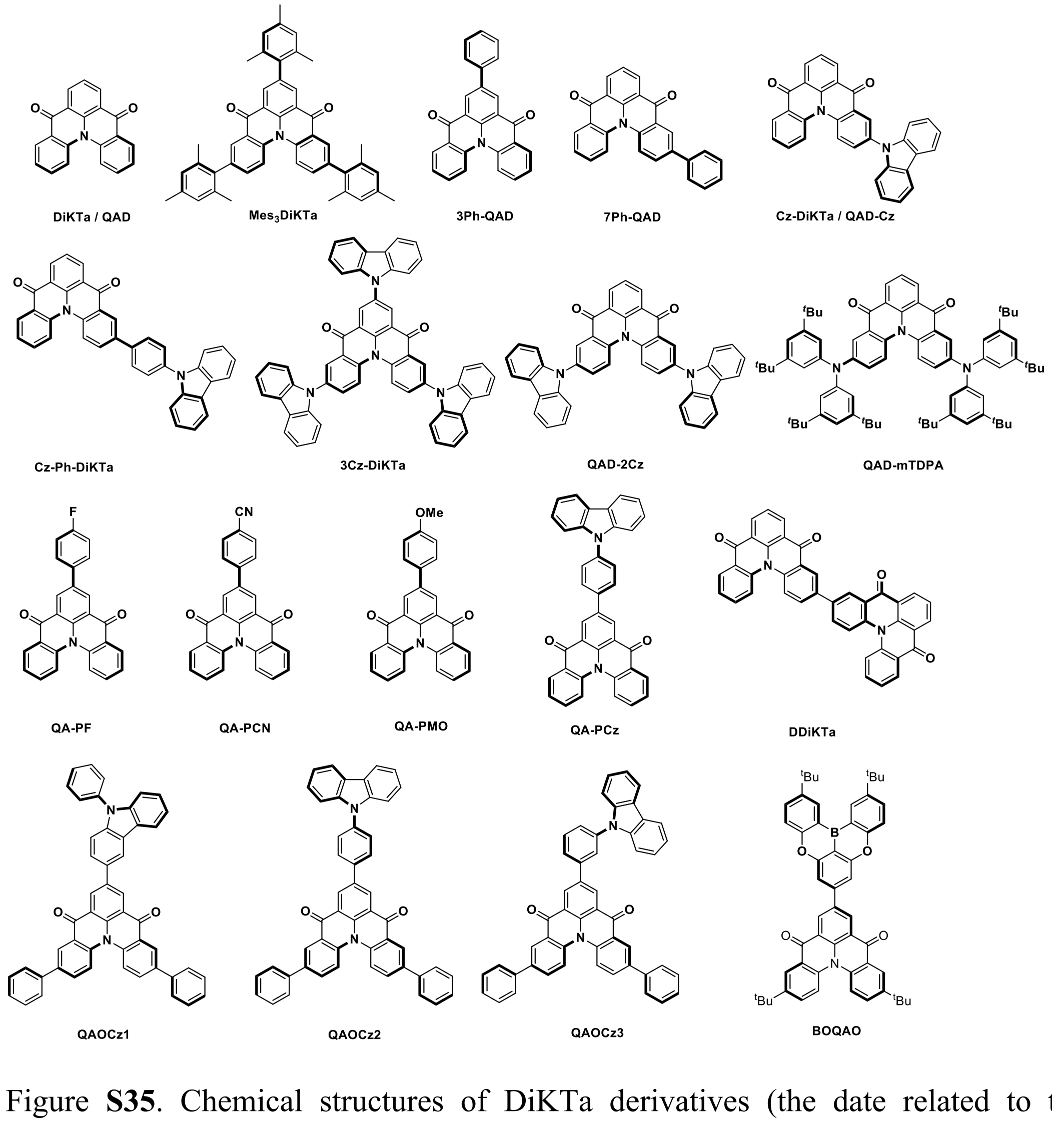

Figure **S35**. Chemical structures of DiKTa derivatives (the date related to these

structures are summarised in Table **S9**).

References:

[1] N. G. Connelly, W. E. Geiger, *Chem. Rev.* **1996**, *96*, 877-910.

[2] a) J. Demasa, G. Crosby, *J. Chem. Phys* **1968**, *48*, 4726; b) G. A. D. Crosby, J.N., *The Journal of Physical Chemistry* **1971**, *75*, 991-1024.

[3] M. Fischer, J. Georges, *Chem. Phys. Lett.* **1996**, *260*, 115-118.

[4] N. C. Greenham, I. D. W. Samuel, G. R. Hayes, R. T. Phillips, Y. A. R. R. Kessener, S. C. Moratti, A. B. Holmes, R. H. Friend, *Chem. Phys. Lett.* **1995**, *241*, 89-96.

[5] F. B. Dias, T. J. Penfold, A. P. Monkman, *Methods Appl. Fluoresc.* **2017**, *5*, 012001.

[6] a) J. Wasey, A. Safonov, I. Samuel, W. Barnes, *Opt. Commun.* **2000**, *183*, 109-121; b) J. Frischeisen, D. Yokoyama, C. Adachi, W. Brütting, *Appl. Physl. Lett.* **2010**, *96*, 29.

[7] T. D. Schmidt, T. Lampe, P. I. Djurovich, M. E. Thompson, W. Brütting, *Phys. Rev. Appl.* **2017**, *8*, 037001.

[8] S. Wu, W. Li, K. Yoshida, D. Hall, S. Madayanad Suresh, T. Sayner, J. Gong, D. Beljonne, Y. Olivier, I. D. W. Samuel, E. Zysman-Colman, *ACS Appl. Mater. Interfaces* **2022**, *14*, 22341-22352.

[9] a) K. A. Neyts, *J. Opt. Soc. Am. A* **1998**, *15*, 962-971; b) S. Nowy, B. C. Krummacher, J. Frischeisen, N. A. Reinke, W. Brütting, *J. Appl. Phys.* **2008**, *104*, 123109; c) M. Furno, R. Meerheim, S. Hofmann, B. Lüssem, K. Leo, *Phys. Rev. B* **2012**, *85*, 115205.

[10] G. W. T. M. J. Frisch, H. B. Schlegel, G. E. Scuseria, M. A. Robb, J. R. Cheeseman, G. Scalmani, V. Barone, B. Mennucci, G. A. Petersson, H. Nakatsuji, M. Caricato, X. Li, H. P. Hratchian, A. F. Izmaylov, J. Bloino, G. Zheng, J. L. Sonnenberg, M. Hada, M. Ehara, K. Toyota, R. Fukuda, J. Hasegawa, M. Ishida, T. Nakajima, Y. Honda, O. Kitao, H. Nakai, T. Vreven, J. A. Montgomery Jr., J. E. Peralta, F. Ogliaro, M. Bearpark, J. J. Heyd, E. Brothers, K. N. Kudin, V. N. Staroverov, R. Kobayashi, J. Normand, K. Raghavachari, A. Rendell, J. C. Burant, S. S. Iyengar, J. Tomasi, M. Cossi, N. Rega, J. M. Millam, M. Klene, J. E. Knox, J. B. Cross, V. Bakken, C. Adamo, J. Jaramillo, R. Gomperts, R. E. Stratmann, O. Yazyev, A. J. Austin, R. Cammi, C. Pomelli, J. W. Ochterski, R. L. Martin, K. Morokuma, V. G. Zakrzewski, G. A. Voth, P. Salvador, J. J. Dannenberg, S. Dapprich, A. D. Daniels, Ö. Farkas, J. B. Foresman, J. V. Ortiz, J. Cioslowski, D. J. Fox, , *Gaussian Inc., Wallingford, CT,* **2009**.

[11] C. Adamo, V. Barone, *J. Chem. Phys.* **1999**, *110*, 6158-6170.

[12] T. H. Dunning Jr, *J. Chem. Phys.* **1989**, *90*, 1007-1023.

[13] a) S. Grimme, *Chem. Phys. Lett.* **1996**, *259*, 128-137; b) S. Hirata, M. Head-Gordon, *Chem. Phys. Lett.* **1999**, *314*, 291-299.

[14] G. A. Petersson, M. A. Al‐Laham, *J. Chem. Phys.* **1991**, *94*, 6081-6090.

[15] K. Momma, F. Izumi, *J. Appl. Crystallogr.* **2011**, *44*, 1272-1276.

[16] in *CrystalClear-SM Expert v2.1. Rigaku Americas, The Woodlands, Texas, USA, and Rigaku Corporation, Tokyo, Japan, 2015.*

[17] a) *CrysAlisPro v1.171.40.14a and 1.171.41.93a.* ; b) *Rigaku Oxford Diffraction, Rigaku Corporation, Oxford, U.K., 2018-2020.*

[18] G. M. Sheldrick, *Acta Crystallogr. A* **2015**, *71*, 3-8.

[19] G. M. Sheldrick, *Acta Crystallogr. C* **2015**, *71*, 3-8.

[20] A. L. Spek, *Acta Crystallogr. C* **2015**, *71*, 9-18.

[21] A. L. Spek, *Acta Crystallogr. D* **2009**, *65*, 148-155.

[22] O. V. Dolomanov, L. J. Bourhis, R. J. Gildea, J. A. Howard, H. Puschmann, *J. Appl. Crystallogr.* **2009**, *42*, 339-341.

[23] S. Madayanad Suresh, D. Hall, D. Beljonne, Y. Olivier, E. Zysman‐Colman, *Adv. Funct. Mater.* **2020**, *30*, 1908677.

[24] N. G. Connelly, W. E. Geiger, *Chemical Reviews* **1996**, *96*, 877-910.

[25] J. Mooney, P. Kambhampati, *J. Phys. Chem. Lett.* **2013**, *4*, 3316-3318.

[26] a) K. Masui, H. Nakanotani, C. Adachi, *Org. Electron.* **2013**, *14*, 2721-2726; b) Y. Tsuchiya, S. Diesing, F. Bencheikh, Y. Wada, P. L. Dos Santos, H. Kaji, E. Zysman-

Colman, I. D. Samuel, C. Adachi, *J. Phys. Chem. A* **2021**, *125*, 8074-8089.
[27] D. Hall, S. M. Suresh, P. L. dos Santos, E. Duda, S. Bagnich, A. Pershin, P. Rajamalli, D. B. Cordes, A. M. Z. Slawin, D. Beljonne, A. Köhler, I. D. W. Samuel, Y. Olivier, E. Zysman-Colman, *Adv. Opt. Mater.* **2020**, *8*, 1901627.
[28] Y. Yuan, X. Tang, X.-Y. Du, Y. Hu, Y.-J. Yu, Z.-Q. Jiang, L.-S. Liao, S.-T. Lee, *Adv. Opt. Mater.* **2019**, *7*, 1801536.
[29] X. Li, Y.-Z. Shi, K. Wang, M. Zhang, C.-J. Zheng, D.-M. Sun, G.-L. Dai, X.-C. Fan, D.-Q. Wang, W. Liu, *ACS Appl. Mater. Interfaces* **2019**, *11*, 13472-13480.
[30] F. Huang, K. Wang, Y.-Z. Shi, X.-C. Fan, X. Zhang, J. Yu, C.-S. Lee, X.-H. Zhang, *ACS Appl. Mater. Interfaces* **2021**, *13*, 36089-36097.
[31] D. Sun, S. M. Suresh, D. Hall, M. Zhang, C. Si, D. B. Cordes, A. M. Z. Slawin, Y. Olivier, X. Zhang, E. Zysman-Colman, *Mater. Chem. Front.* **2020**, *4*, 2018-2022.
[32] X. Qiu, G. Tian, C. Lin, Y. Pan, X. Ye, B. Wang, D. Ma, D. Hu, Y. Luo, Y. Ma, *Adv. Opt. Mater.* **2021**, *9*, 2001845.
[33] J.-F. Liu, S.-N. Zou, X. Chen, S.-Y. Yang, Y.-J. Yu, M.-K. Fung, Z.-Q. Jiang, L.-S. Liao, *Mater. Chem. Front.* **2022**, *6*, 966-972.
[34] Y.-J. Yu, S.-N. Zou, C.-C. Peng, Z.-Q. Feng, Y.-K. Qu, S.-Y. Yang, Z.-Q. Jiang, L.-S. Liao, *J. Mater. Chem. C* **2022**, *10*, 4941-4946.
