# Supplementary material for: Highly Efficient Green and Red Narrowband Emissive Organic Light‐Emitting Diodes Employing Multi‐Resonant Thermally Activated Delayed Fluorescence Emitters
Source: Angew Chem Int Ed Engl. 2022 Nov 23;61(52):e202213697. doi: 10.1002/anie.202213697 (PMC10100094; doi:10.1002/anie.202213697)

checkCIF/PLATON report

Structure factors have been supplied for datablock(s) 3DPA-DiKTa, 3TPA-DiKTa

THIS REPORT IS FOR GUIDANCE ONLY. IF USED AS PART OF A REVIEW PROCEDURE FOR PUBLICATION, IT SHOULD NOT REPLACE THE EXPERTISE OF AN EXPERIENCED CRYSTALLOGRAPHIC REFEREE.

No syntax errors found. CIF dictionary Interpreting this report

Datablock: 3TPA-DiKTa

Bond precision: C-C = 0.0123 A Wavelength=1.54184

Cell:	a=10.9144(11)	b=17.2054(11)	c=17.3910(12)
	alpha=66.543(6)	beta=80.988(7)	gamma=76.734(7)
Temperature:	173 K		

	Calculated	Reported
Volume	2908.2(4)	2908.2(4)
Space group	P -1	P -1
Hall group	-P 1	-P 1
Moiety formula	C74 H50 N4 O2 [+ solvent]	C74 H50 N4 O2
Sum formula	C74 H50 N4 O2 [+ solvent]	C74 H50 N4 O2
Mr	1027.18	1027.18
Dx,g cm-3	1.173	1.173
Z	2	2
Mu (mm-1)	0.549	0.549
F000	1076.0	1076.0
F000’	1078.96	
h,k,lmax	13,20,20	13,20,20
Nref	10634	10312
Tmin,Tmax	0.961,0.978	0.353,1.000
Tmin’	0.858	

Correction method= # Reported T Limits: Tmin=0.353 Tmax=1.000
AbsCorr = MULTI-SCAN

Data completeness= 0.970 Theta(max)= 68.240

R(reflections)= 0.1756(4104) wR2(reflections)= 0.5199(10312)

S = 1.504 Npar= 860

The following ALERTS were generated. Each ALERT has the format
test-name_ALERT_alert-type_alert-level.
Click on the hyperlinks for more details of the test.

Alert level A

PLAT084_ALERT_3_A High wR2 Value (i.e. > 0.25) .... 0.52 Report

Author Response: Crystal diffracts weakly, no diffraction above 1.09 A.

PLAT410_ALERT_2_A Short Intra H...H Contact H3 ..H19 . 1.62 Ang.
x,y,z = 1_555 Check

Author Response: Unmodelled disorder in this part of the molecule. Attempts to extend disorder model unsuccessful.

Alert level B

PLAT082_ALERT_2_B High R1 Value .... 0.18 Report

Author Response: Crystal diffracts weakly, no diffraction above 1.09 A.

PLAT097_ALERT_2_B Large Reported Max. (Positive) Residual Density 1.05 eA-3

Author Response: Probably part of diffuse solvent in pores, in too close proximity to minor component of a disordered ring to be removed during SQUEEZE.

PLAT230_ALERT_2_B Hirshfeld Test Diff for C13 --C14 . 7.7 s.u.

Author Response: Unmodelled disorder in this part of the molecule. Attempts to extend disorder model unsuccessful.

PLAT340_ALERT_3_B Low Bond Precision on C-C Bonds .... 0.01233 Ang.

Author Response: Lower than ideal data quality arising from weak high angle diffraction and extent of disorder within the molecule.

Alert level C

DIFMX02_ALERT_1_C The maximum difference density is > 0.1*ZMAX*0.75
The relevant atom site should be identified.

```
PLAT026_ALERT_3_C Ratio Observed / Unique Reflections (too) Low ..        40% Check
PLAT029_ALERT_3_C _diffrn_measured_fraction_theta_full value Low .      0.978 Why?
PLAT094_ALERT_2_C Ratio of Maximum / Minimum Residual Density ....       2.92 Report
PLAT230_ALERT_2_C Hirshfeld Test Diff for    O14      --C14      .        7.0 s.u.
```

Author Response: Unmodelled disorder in this part of the molecule. Attempts to extend disorder model unsuccessful.

```
PLAT234_ALERT_4_C Large Hirshfeld Difference N28      --C35A     .       0.24 Ang.
PLAT234_ALERT_4_C Large Hirshfeld Difference N47      --C48A     .       0.18 Ang.
PLAT234_ALERT_4_C Large Hirshfeld Difference N66      --C67      .       0.22 Ang.
PLAT234_ALERT_4_C Large Hirshfeld Difference N66      --C73      .       0.24 Ang.
PLAT234_ALERT_4_C Large Hirshfeld Difference C3       --C4       .       0.16 Ang.
PLAT234_ALERT_4_C Large Hirshfeld Difference C18      --C19      .       0.17 Ang.
PLAT234_ALERT_4_C Large Hirshfeld Difference C29      --C30      .       0.18 Ang.
PLAT234_ALERT_4_C Large Hirshfeld Difference C29      --C34      .       0.19 Ang.
PLAT234_ALERT_4_C Large Hirshfeld Difference C31      --C32      .       0.21 Ang.
PLAT234_ALERT_4_C Large Hirshfeld Difference C39      --C40      .       0.16 Ang.
PLAT234_ALERT_4_C Large Hirshfeld Difference C49      --C50      .       0.22 Ang.
PLAT234_ALERT_4_C Large Hirshfeld Difference C67      --C72      .       0.23 Ang.
PLAT234_ALERT_4_C Large Hirshfeld Difference C68      --C69      .       0.18 Ang.
PLAT234_ALERT_4_C Large Hirshfeld Difference C71      --C72      .       0.23 Ang.
PLAT234_ALERT_4_C Large Hirshfeld Difference C74      --C75      .       0.17 Ang.
PLAT241_ALERT_2_C High   'MainMol' Ueq as Compared to Neighbors of         C3 Check
PLAT241_ALERT_2_C High   'MainMol' Ueq as Compared to Neighbors of         C6 Check
PLAT241_ALERT_2_C High   'MainMol' Ueq as Compared to Neighbors of        C15 Check
PLAT241_ALERT_2_C High   'MainMol' Ueq as Compared to Neighbors of        C16 Check
PLAT241_ALERT_2_C High   'MainMol' Ueq as Compared to Neighbors of        C18 Check
PLAT241_ALERT_2_C High   'MainMol' Ueq as Compared to Neighbors of        C19 Check
PLAT241_ALERT_2_C High   'MainMol' Ueq as Compared to Neighbors of        C26 Check
PLAT241_ALERT_2_C High   'MainMol' Ueq as Compared to Neighbors of        C27 Check
PLAT242_ALERT_2_C Low    'MainMol' Ueq as Compared to Neighbors of         C2 Check
PLAT242_ALERT_2_C Low    'MainMol' Ueq as Compared to Neighbors of         C5 Check
PLAT242_ALERT_2_C Low    'MainMol' Ueq as Compared to Neighbors of        C20 Check
PLAT250_ALERT_2_C Large U3/U1 Ratio for Average U(i,j) Tensor ....        2.1 Note
PLAT260_ALERT_2_C Large Average Ueq of Residue Including       O8      0.133 Check
PLAT334_ALERT_2_C Small <C-C> Benzene Dist.   C15      -C20      .       1.37 Ang.
PLAT906_ALERT_3_C Large K Value in the Analysis of Variance ......     24.414 Check
PLAT906_ALERT_3_C Large K Value in the Analysis of Variance ......      4.678 Check
PLAT906_ALERT_3_C Large K Value in the Analysis of Variance ......      2.573 Check
PLAT911_ALERT_3_C Missing FCF Refl Between Thmin & STh/L=    0.600        235 Report
```

Alert level G

```
PLAT002_ALERT_2_G Number of Distance or Angle Restraints on AtSite         25 Note
PLAT003_ALERT_2_G Number of Uiso or Uij Restrained non-H Atoms ...         94 Report
PLAT072_ALERT_2_G SHELXL First  Parameter in WGHT  Unusually Large       0.20 Report
PLAT172_ALERT_4_G The CIF-Embedded .res File Contains DFIX Records          4 Report
PLAT178_ALERT_4_G The CIF-Embedded .res File Contains SIMU Records          5 Report
PLAT187_ALERT_4_G The CIF-Embedded .res File Contains RIGU Records          3 Report
PLAT301_ALERT_3_G Main Residue  Disorder .............(Resd  1  )         54% Note
PLAT410_ALERT_2_G Short Intra H...H Contact  H16      ..H61      .       2.04 Ang.
                                                      x,y,z  =      1_555 Check
```

Author Response: Unmodelled disorder in this part of the molecule. Attempts to extend disorder model unsuccessful.

```
PLAT410_ALERT_2_G Short Intra H...H Contact  H18      ..H65      .       2.00 Ang.
                                                       x,y,z  =      1_555 Check
```

Author Response: Unmodelled disorder in this part of the molecule. Attempts to extend disorder model unsuccessful.

```
PLAT605_ALERT_4_G Largest Solvent Accessible VOID in the Structure        115 A**3
PLAT811_ALERT_5_G No ADDSYM Analysis: Too Many Excluded Atoms ....          ! Info
PLAT860_ALERT_3_G Number of Least-Squares Restraints ............        1360 Note
PLAT869_ALERT_4_G ALERTS Related to the Use of SQUEEZE  Suppressed          ! Info
PLAT912_ALERT_4_G Missing # of FCF Reflections Above STh/L=  0.600         86 Note
PLAT933_ALERT_2_G Number of HKL-OMIT Records in Embedded .res File          3 Note
PLAT941_ALERT_3_G Average HKL Measurement Multiplicity ..........         2.9 Low
PLAT978_ALERT_2_G Number C-C Bonds with Positive Residual Density.          3 Info
PLAT992_ALERT_5_G Repd & Actual _reflns_number_gt Values Differ by          3 Check
```

```
   2 ALERT level A = Most likely a serious problem - resolve or explain
   4 ALERT level B = A potentially serious problem, consider carefully
  38 ALERT level C = Check. Ensure it is not caused by an omission or oversight
  18 ALERT level G = General information/check it is not something unexpected

   1 ALERT type 1 CIF construction/syntax error, inconsistent or missing data
  27 ALERT type 2 Indicator that the structure model may be wrong or deficient
  11 ALERT type 3 Indicator that the structure quality may be low
  21 ALERT type 4 Improvement, methodology, query or suggestion
   2 ALERT type 5 Informative message, check
```

Datablock: 3DPA-DiKTa

```
Bond precision:   C-C = 0.0023 A           Wavelength=1.54184

Cell:           a=11.0801(2)       b=12.5559(2)      c=17.2089(3)
                alpha=88.893(1)    beta=89.488(1)    gamma=83.638(1)
Temperature:    173 K
```

	Calculated	Reported
Volume	2378.87(7)	2378.87(7)
Space group	P -1	P -1
Hall group	-P 1	-P 1
Moiety formula	C56 H38 N4 O2, C H2 Cl2 [+ solvent]	C56 H38 N4 O2, C H2 Cl2
Sum formula	C57 H40 Cl2 N4 O2 [+ solvent]	C57 H40 Cl2 N4 O2
Mr	883.83	883.83
Dx,g cm-3	1.234	1.234
Z	2	2
Mu (mm-1)	1.592	1.592
F000	920.0	920.0
F000'	923.84	
h,k,lmax	13,15,21	13,15,21
Nref	9863	9419
Tmin,Tmax	0.926,0.984	0.861,1.000
Tmin'	0.788	

Correction method= # Reported T Limits: Tmin=0.861 Tmax=1.000
AbsCorr = MULTI-SCAN

Data completeness= 0.955 Theta(max)= 75.485

R(reflections)= 0.0462(8043) wR2(reflections)= 0.1384(9419)

S = 1.058 Npar= 641

The following ALERTS were generated. Each ALERT has the format
test-name_ALERT_alert-type_alert-level.
Click on the hyperlinks for more details of the test.

Alert level C

```
PLAT911_ALERT_3_C Missing FCF Refl Between Thmin & STh/L=    0.600        154 Report
PLAT918_ALERT_3_C Reflection(s) with I(obs) much Smaller I(calc) .          1 Check
PLAT934_ALERT_3_C Number of (Iobs-Icalc)/Sigma(W) > 10 Outliers ..          1 Check
```

Alert level G

```
PLAT003_ALERT_2_G Number of Uiso or Uij Restrained non-H Atoms ...         12 Report
PLAT154_ALERT_1_G The s.u.'s on the Cell Angles are Equal ..(Note)      0.001 Degree
PLAT178_ALERT_4_G The CIF-Embedded .res File Contains SIMU Records          1 Report
PLAT187_ALERT_4_G The CIF-Embedded .res File Contains RIGU Records          1 Report
PLAT301_ALERT_3_G Main Residue  Disorder ..............(Resd  1  )        10% Note
PLAT605_ALERT_4_G Largest Solvent Accessible VOID in the Structure        266 A**3
PLAT720_ALERT_4_G Number of Unusual/Non-Standard Labels ..........          3 Note
PLAT860_ALERT_3_G Number of Least-Squares Restraints .............        225 Note
```

```
PLAT869_ALERT_4_G ALERTS Related to the Use of SQUEEZE  Suppressed          ! Info
PLAT912_ALERT_4_G Missing # of FCF Reflections Above STh/L=  0.600        291 Note
PLAT913_ALERT_3_G Missing # of Very Strong Reflections in FCF ....          1 Note
PLAT941_ALERT_3_G Average HKL Measurement Multiplicity ..........         3.0 Low
PLAT978_ALERT_2_G Number C-C Bonds with Positive Residual Density.          8 Info
PLAT992_ALERT_5_G Repd & Actual _reflns_number_gt Values Differ by          3 Check
```

```
   0 ALERT level A = Most likely a serious problem - resolve or explain
   0 ALERT level B = A potentially serious problem, consider carefully
   3 ALERT level C = Check. Ensure it is not caused by an omission or oversight
  14 ALERT level G = General information/check it is not something unexpected

   1 ALERT type 1 CIF construction/syntax error, inconsistent or missing data
   2 ALERT type 2 Indicator that the structure model may be wrong or deficient
   7 ALERT type 3 Indicator that the structure quality may be low
   6 ALERT type 4 Improvement, methodology, query or suggestion
   1 ALERT type 5 Informative message, check
```

It is advisable to attempt to resolve as many as possible of the alerts in all categories. Often the minor alerts point to easily fixed oversights, errors and omissions in your CIF or refinement strategy, so attention to these fine details can be worthwhile. In order to resolve some of the more serious problems it may be necessary to carry out additional measurements or structure refinements. However, the purpose of your study may justify the reported deviations and the more serious of these should normally be commented upon in the discussion or experimental section of a paper or in the "special_details" fields of the CIF. checkCIF was carefully designed to identify outliers and unusual parameters, but every test has its limitations and alerts that are not important in a particular case may appear. Conversely, the absence of alerts does not guarantee there are no aspects of the results needing attention. It is up to the individual to critically assess their own results and, if necessary, seek expert advice.

Publication of your CIF in IUCr journals

A basic structural check has been run on your CIF. These basic checks will be run on all CIFs submitted for publication in IUCr journals (*Acta Crystallographica*, *Journal of Applied Crystallography*, *Journal of Synchrotron Radiation*); however, if you intend to submit to *Acta Crystallographica Section C* or *E* or *IUCrData*, you should make sure that full publication checks are run on the final version of your CIF prior to submission.

Publication of your CIF in other journals

Please refer to the *Notes for Authors* of the relevant journal for any special instructions relating to CIF submission.

PLATON version of 18/05/2022; check.def file version of 17/05/2022

Datablock 3TPA-DiKTa - ellipsoid plot

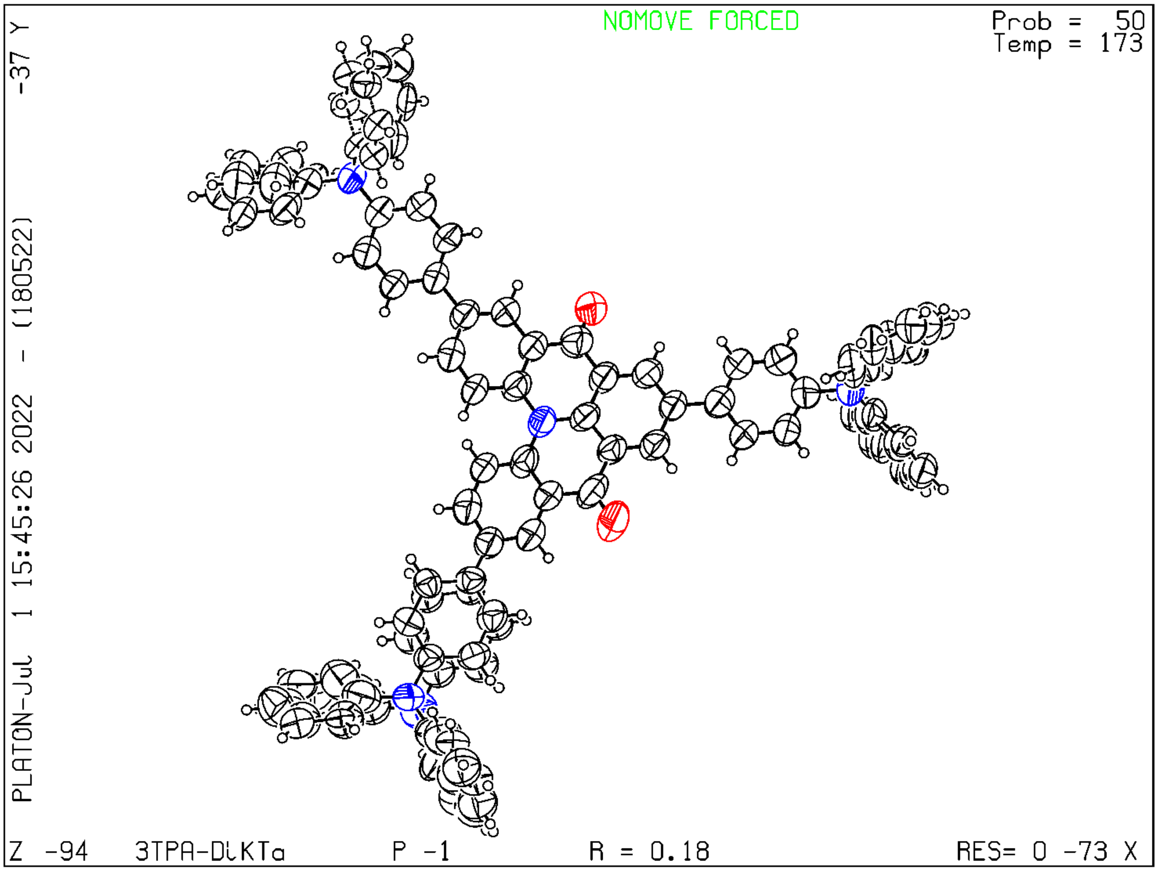

Datablock 3DPA-DiKTa - ellipsoid plot

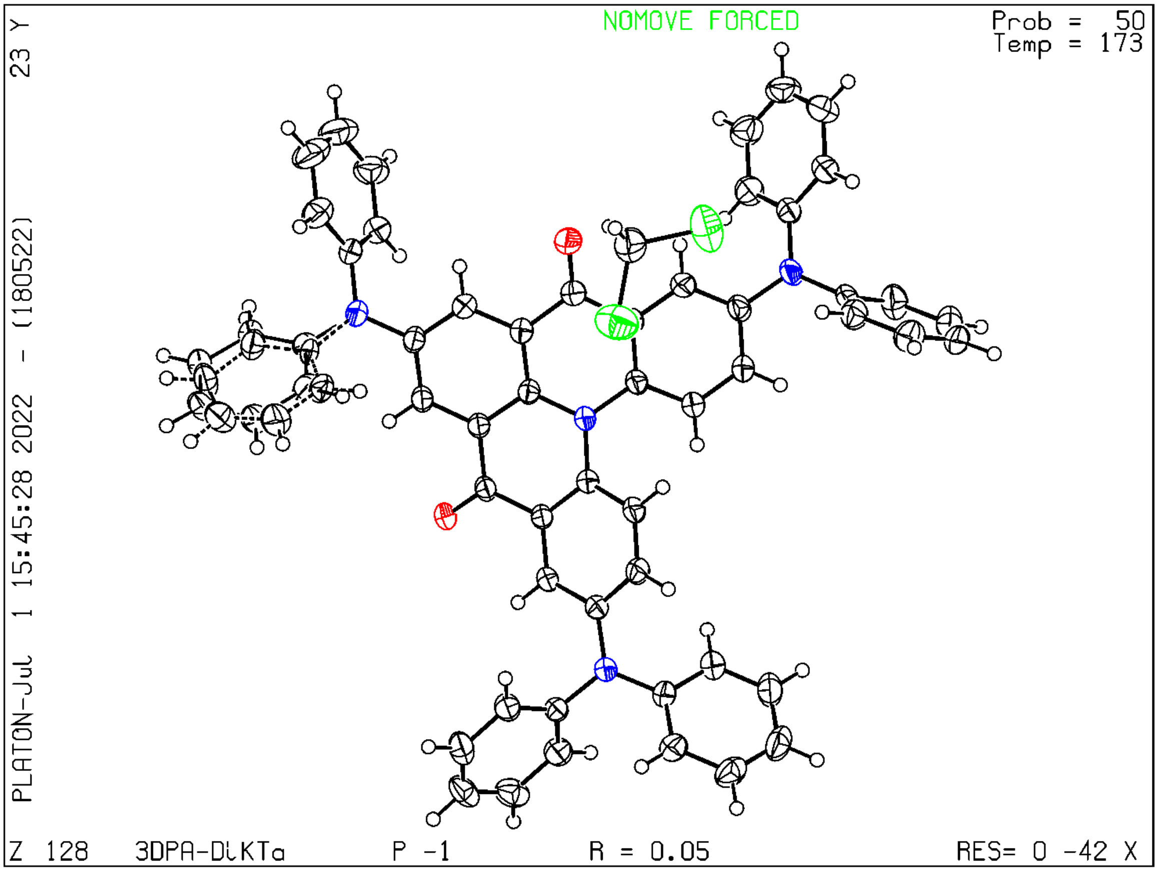

Supplement: Supplementary file 2 — Supporting Information [file ANIE-61-0-s001.pdf]
